# Batch-to-Flow Translation of β-Cyclodextrin Polymer Adsorption for Emerging Contaminant Removal: Hydrodynamic and Operational Validation

**DOI:** 10.3390/polym18182292

**Published:** 2026-09-19

**Authors:** Antonio Tomás Hernández Cegarra, Teresa Gómez-Morte, José Antonio Pellicer, María Isabel Rodríguez-López, Nuria Vela, Ángel Gil-Izquierdo, Estrella Núñez-Delicado, José Antonio Gabaldón

**Affiliations:** 1 Molecular Recognition and Encapsulation Research Group (REM), Health Sciences Department, Universidad Católica de Murcia (UCAM), Campus de los Jerónimos 135, E-30107 Guadalupe, Spain; athernandez@alu.ucam.edu (A.T.H.C.); tgomez@ucam.edu (T.G.-M.); japellicer@ucam.edu (J.A.P.); mirodriguez@ucam.edu (M.I.R.-L.); enunez@ucam.edu (E.N.-D.); 2 Applied Technology Group to Environmental Health, Universidad Católica de Murcia (UCAM), Campus de los Jerónimos 135, E-30107 Guadalupe, Spain; nvela@ucam.edu; 3 Research Group on Quality, Safety, and Bioactivity of Plant-Derived Foods, Department of Food Science and Technology, Centro de Edafología y Biología Aplicada del Segura–Consejo Superior de Investigaciones Científicas (CEBAS-CSIC), Campus de Espinardo 25, E-30100 Murcia, Spain; angelgil@cebas.csic.es

**Keywords:** β-cyclodextrin-epichlorohydrin polymer, continuous-flow adsorption, upflow mobile bed, emerging contaminants, competitive adsorption, wastewater matrix, regeneration, scale-up

## Abstract

Translating adsorption performance from batch experiments to continuous-flow operation is a key step toward practical water-treatment applications. In this study, a laboratory-scale continuous adsorption system based on a water-insoluble β-cyclodextrin-epichlorohydrin (β-CD-EPI) polymer was validated from hydrodynamic, adsorptive, and operational perspectives. Downflow operation caused progressive bed compaction and excessive pressure development, whereas a 90 mm column operated in upflow mobile-bed mode, with visually observed bed expansion, showed comparatively stable pressure-drop behavior at superficial linear velocities below approximately 12 m h^−1^. Under these controlled high-loading conditions, removal was strongly contaminant-dependent: cyproconazole exceeded 90%, acetaminophen reached 72–77%, hydrochlorothiazide reached 40–65%, ciprofloxacin reached 24–50%, and furosemide remained below 30%. The relative performance for furosemide and hydrochlorothiazide differed from that predicted by previous batch-derived adsorption parameters, demonstrating that batch results cannot be directly extrapolated to dynamic operation. Competitive adsorption in binary and ternary mixtures reduced contaminant removal, while cyproconazole removal decreased from >90% in tap water to 48–55% in secondary-treated wastewater, demonstrating the relevance of the aqueous matrix under the tested continuous-flow conditions. Operational screening tests showed that desorption with 220 mM acetate buffer at pH 4.0 recovered >80% of the retained cyproconazole within 10 min in the tested sequence, followed by a two-stage rinse that restored the operational pH. These preliminary conditions require confirmation through replicated adsorption–desorption cycles under continuous-flow operation. These results identify laboratory-scale hydrodynamic, adsorption, matrix, and regeneration considerations that require confirmation through fixed-condition, long-term testing during subsequent process development.

## 1. Introduction

Contaminants of emerging concern (CECs), including pharmaceuticals, personal care products, pesticides, endocrine-disrupting compounds, biocides, and a wide range of industrial chemicals, are widely detected in aquatic environments and wastewater streams worldwide, typically at trace concentrations ranging from the ng/L to the µg/L level [1]. Their continuous release into the aquatic environment is of concern because many CECs are biologically active, persistent, or incompletely removed during wastewater treatment, and some may exert adverse effects on aquatic organisms even at low concentrations [2]. Municipal wastewater treatment plants (WWTPs) therefore represent an important pathway for the discharge of CECs into receiving waters, since conventional treatment processes were primarily designed to remove suspended solids, biodegradable organic matter, and nutrients rather than structurally diverse organic micropollutants [3]. Consequently, removal efficiencies vary widely among compounds according to their physicochemical properties and the treatment processes applied, and measurable concentrations may remain in treated effluents [4]. This issue has acquired additional regulatory relevance in Europe following the adoption of Directive (EU) 2024/3019 on urban wastewater treatment, which explicitly incorporates quaternary treatment to reduce a broad spectrum of micropollutants in urban wastewater [5]. These developments reinforce the need for advanced treatment technologies combining high contaminant removal with operational robustness, scalability, and compatibility with continuous wastewater-treatment processes [6].

Among the technologies available for CEC removal, adsorption is particularly attractive as a polishing treatment because of its operational simplicity, high removal potential, modularity, and adaptability to existing treatment trains. Within this context, cyclodextrin (CD)-based polymers have emerged as a versatile class of adsorbent materials for the removal of organic contaminants from water and wastewater [7]. Cyclodextrins are cyclic oligosaccharides characterized by a hydrophilic external surface and a relatively hydrophobic internal cavity capable of accommodating organic guest molecules through host–guest inclusion when molecular size, geometry, and chemical affinity are favorable. Cross-linking cyclodextrins into water-insoluble polymeric networks overcomes their intrinsic water solubility while retaining accessible inclusion cavities and generating a three-dimensional polymer matrix that provides additional interaction domains for pollutant retention [8]. These networks can therefore combine host–guest complexation with interactions involving the polymer framework, including hydrophobic interactions, hydrogen bonding, and, depending on polymer composition and solution chemistry, electrostatic interactions [8]. Their insolubility also facilitates physical retention within treatment systems and enables their potential regeneration and reuse over successive adsorption–desorption cycles [9].

Among these materials, β-cyclodextrin-epichlorohydrin (β-CD-EPI) polymers represent a particularly attractive class of cross-linked CD adsorbents because epichlorohydrin produces a hydrated, three-dimensional network containing accessible β-CD cavities together with hydroxyl-rich polymer domains capable of interacting with chemically diverse solutes. β-CD-EPI polymers have previously demonstrated the ability to remove a broad range of pharmaceutical and other organic contaminants under batch conditions, although adsorption efficiency is strongly compound-dependent [10]. More broadly, recent research has substantially expanded the structural diversity, porosity, functionalization, and adsorption performance of CD-based polymers [11]. Nevertheless, the development of these materials for water treatment remains dominated by synthesis, structural characterization, and batch-scale adsorption studies, whereas their translation into stable continuous treatment systems is still comparatively underexplored. This represents an important step because the properties governing adsorption under equilibrium batch conditions do not necessarily determine performance once the polymer is incorporated into a continuously operated adsorbent bed.

The versatility of EPI-cross-linked cyclodextrin networks has also been demonstrated in previous studies from our group using different CD architectures for aqueous contaminant removal. A water-insoluble β-CD-EPI polymer was successfully applied to the adsorption of Direct Blue 78 from an aqueous model system, with its adsorption behavior characterized through kinetic, equilibrium, and thermodynamic analyses [12]. Subsequently, γ-CD-EPI and hydroxypropyl-γ-CD-EPI polymers were developed as adsorbents for Direct Red 83:1, demonstrating that EPI cross-linking can generate functional water-insoluble adsorbents from different CD derivatives and that their adsorption behavior depends on the resulting polymer architecture [13]. Together, these studies established the versatility of CD-EPI networks as functional materials for aqueous pollutant removal and provided a material-level basis for progressing from batch-scale adsorption characterization toward operational validation under continuous-flow conditions.

Batch experiments remain essential for determining adsorption equilibrium, kinetics, selectivity, and the influence of solution chemistry, and the resulting parameters provide a valuable basis for adsorbent evaluation and preliminary process design [14]. Under continuous-flow conditions, however, adsorption performance is additionally influenced by contact time, flow velocity, bed geometry, external and intraparticle mass transfer, and progressive adsorbent loading, meaning that equilibrium capacity alone cannot reliably predict reactor performance [14]. This limitation has recently been demonstrated for β-CD-based adsorbents, for which batch sorption kinetics did not fully reproduce column behavior under flow-through conditions [15]. For hydrated polymeric adsorbents, an additional consideration is the physical response of the polymer bed to liquid flow. Swelling, deformation, compression or expansion, particle entrainment, channeling, and pressure development may ultimately determine whether continuous operation is feasible. Reactor configuration is therefore particularly important for cross-linked polymers that undergo substantial hydration, because fixed- and fluidized-bed operation impose markedly different hydrodynamic conditions and can strongly affect bed porosity, hydraulic resistance, solid–liquid contact, and mass-transfer behavior [16]. Although continuous treatment using CD-based adsorbents has been demonstrated at pilot scale, including the removal of micropollutants from treated municipal wastewater using a β-CD bead polymer under fluidized conditions [17], systematic studies integrating polymer-bed hydrodynamics with adsorption performance remain limited.

A further challenge arises when CD-based adsorbents are transferred from simplified single-solute systems to complex aqueous matrices. Real wastewater contains numerous micropollutants that may interact simultaneously with a finite number of accessible cyclodextrin cavities and polymer-network domains, thereby modifying apparent affinity and selectivity through competitive adsorption [15]. Dissolved organic matter, inorganic ions, and other wastewater constituents may also interfere with adsorption by competing for interaction sites, modifying electrostatic interactions, or hindering solute transport within the polymer network [18]. The magnitude of these effects depends on both adsorbent chemistry and the physicochemical characteristics of the target contaminant. Recent studies using full- and pilot-scale adsorption systems similarly indicate that competitive adsorption and wastewater-matrix effects can substantially reduce the direct transferability of parameters obtained from simplified laboratory experiments to realistic treatment conditions [19]. Consequently, meaningful validation of CD-based polymer adsorbents should progressively move from single-contaminant model solutions toward multicomponent systems and real wastewater matrices while maintaining controlled hydrodynamic conditions.

In our previous work, a stepwise strategy was developed to translate β-CD polymer adsorption data from batch experiments into the engineering design of a laboratory-scale continuous-flow prototype [20]. Using an insoluble β-CD-EPI polymer, furosemide and hydrochlorothiazide were employed as model pharmaceuticals to determine adsorption kinetics, equilibrium behavior, and physicochemical parameters relevant to reactor design. These adsorption parameters, together with polymer properties including swelling, density, and particle size, were integrated with engineering criteria such as adsorbent volume, bed depth, contact time, flow rate, regeneration, and rinsing requirements to dimension the prototype [20]. The resulting system incorporated a 90 mm diameter adsorption column as the main configuration and an additional 63 mm diameter column for comparative evaluation. However, that work established the batch-derived design and construction of the prototype without experimentally validating either its polymer-bed hydrodynamics or its adsorption performance under continuous-flow conditions. Continuous-flow experiments with contaminated water and wastewater were therefore identified as the necessary next stage in evaluating whether the design parameters derived from batch adsorption could be successfully transferred to dynamic operation [20].

The present study addresses this unresolved step by experimentally validating the β-CD-EPI polymer adsorption system under continuous-flow conditions. First, the effects of column diameter, polymer loading, superficial linear velocity, and flow direction were evaluated to establish hydrodynamically stable operating conditions. The adsorption behavior of the system was then assessed using five CECs with contrasting physicochemical characteristics, furosemide, hydrochlorothiazide, acetaminophen, ciprofloxacin, and cyproconazole, to determine compound-specific performance and examine the transferability of previously obtained batch parameters. System complexity was subsequently increased through binary and ternary contaminant mixtures and the use of secondary-treated WWTP effluent as the aqueous matrix, allowing competitive adsorption and wastewater-matrix effects to be evaluated under the same continuous-flow framework. Finally, continuous-flow desorption and post-desorption rinsing were investigated as prerequisite operational steps for polymer regeneration and reuse. To the best of our knowledge, no previous study has systematically integrated batch-derived process design, hydrated polymer-bed hydrodynamics, compound-specific dynamic adsorption, multicomponent competition, wastewater-matrix effects, and regeneration within a single experimental framework for a β-CD-EPI polymer adsorption system. The results provide a laboratory-scale experimental basis for identifying the operating conditions and additional validation requirements that should be addressed during subsequent process development.

## 2. Materials and Methods

### 2.1. Chemicals, Contaminants, and Aqueous Matrices

β-Cyclodextrin (β-CD; CID 444041) was supplied by AraChem (Tilburg, The Netherlands). Epichlorohydrin (EPI), sodium borohydride, sodium hydroxide, acetone, and acetate buffer were obtained from Sigma-Aldrich (Barcelona, Spain). Standards for furosemide (CAS No. 54-31-9, ≥98% purity), hydrochlorothiazide (CAS No. 58-93-5, ≥98% purity), acetaminophen (CAS No. 103-90-2, ≥99.0% purity), ciprofloxacin (CAS No. 85721-33-1, ≥98% purity), and cyproconazole (CAS No. 94361-06-5, ≥98% purity; PESTANAL^®^ analytical standard) were supplied by Sigma-Aldrich (Barcelona, Spain). All other reagents were of analytical grade.

The five target contaminants were selected to encompass compounds with distinct physicochemical properties and previously observed adsorption responses. Aqueous solutions for the controlled continuous-flow experiments were prepared using tap water at the concentrations specified for each assay. To evaluate the influence of the wastewater matrix, secondary-treated effluent collected from the Cabezo Beaza municipal wastewater treatment plant (Cartagena, southeastern Spain) was used as the aqueous matrix and spiked with cyproconazole prior to continuous-flow treatment.

### 2.2. β-CD-EPI Polymer

A water-insoluble β-CD network cross-linked with epichlorohydrin (β-CD–EPI) was used as the adsorbent throughout this study. The polymer was synthesized in the UCAM laboratory using a β-CD/EPI cross-linking approach consistent with established preparation routes for water-insoluble β-CD-EPI copolymers [21], following the specific formulation previously reported for this material [20]. Briefly, β-CD (24 g) and sodium borohydride (60 mg) were dispersed in distilled water (24 mL) and maintained under stirring at 50 °C for 10 min. Subsequently, 26 mL of a 40% sodium hydroxide solution was added, and the mixture was stirred for a further 5 min. EPI (264 g) was then added dropwise, and the reaction was maintained under continuous stirring for 6 h. The resulting polymer was washed with 100 mL of acetone for 10 min and subsequently dried at 50 °C for 12 h. The dried material was ground and homogenized prior to use in the continuous-flow experiments.

Previously determined physicochemical properties relevant to reactor operation, including swelling behavior, density, and particle characteristics, were used as engineering inputs for the design and dimensioning of the laboratory-scale prototype [20]. Thus, the present study focused on the hydrodynamic and adsorption behavior of the previously developed β-CD-EPI polymer under continuous-flow conditions rather than on its structural characterization.

### 2.3. Laboratory-Scale Continuous-Flow Prototype

Continuous-flow experiments were performed using the laboratory-scale adsorption prototype previously designed and constructed based on batch-derived adsorption parameters and the physicochemical characteristics of the β-CD-EPI polymer [20]. The system consisted of an adsorption-column circuit connected to 50 L feed and collection vessels. Liquid circulation was provided by a self-priming diaphragm pump, while the operating flow rate was regulated by a needle valve and continuously monitored using an in-line flow meter. Pressure was measured immediately upstream and downstream of the adsorption column, enabling the pressure drop across the polymer bed to be monitored during operation. The system also incorporated pH monitoring and sampling points at both the column inlet and outlet.

The prototype accommodated two transparent PVC adsorption columns with nominal diameters of 90 and 63 mm. The 90 mm column was used as the main configuration for the continuous-flow adsorption experiments, whereas the 63 mm column provided an additional configuration for comparative hydraulic and regeneration experiments. Both columns were equipped with polypropylene retaining elements with a nominal opening of 100 µm to prevent loss of adsorbent while allowing passage of the liquid phase. The transparent column walls enabled direct observation of polymer-bed compaction, expansion, and particle movement during operation. The hydraulic circuit allowed the direction of liquid flow to be switched between downflow and upflow operation, permitting comparison between a conventional downflow configuration and an upflow mobile-bed configuration in which bed expansion and particle movement could be visually observed through the transparent column wall. Adsorbent loadings, superficial linear velocities, and nominal contact times used in the hydrodynamic and adsorption experiments are specified in the following sections.

### 2.4. Hydrodynamic Characterization of the Prototype

Prior to the adsorption experiments, the hydrodynamic behavior of the β-CD-EPI polymer bed was evaluated to identify operating conditions compatible with stable continuous-flow operation. Four variables were examined: column configuration, adsorbent loading, superficial linear velocity, and flow direction. Initial experiments were performed in downflow mode using columns with nominal diameters of 63 and 90 mm. The effective internal cross-sectional areas used for the hydraulic calculations were 0.0026 and 0.0056 m^2^ for the nominal 63 and 90 mm columns, respectively. Three β-CD-EPI loadings (235, 470, and 705 g) were evaluated over a superficial linear-velocity range of 4–20 m h^−1^.

The hydraulic response of the adsorbent bed was assessed by continuously monitoring the pressure difference (Δp) between the column inlet and outlet as a function of operating time. Bed compaction, expansion, and particle movement were simultaneously monitored by visual inspection through the transparent column wall. A pressure drop approaching 1 bar was considered the practical operating limit, while the system overpressure protection was activated at approximately 1.5 bar.

Pressure drop was the quantitatively monitored hydrodynamic variable, whereas bed compaction, expansion, and particle movement were assessed qualitatively by visual inspection through the transparent column wall. Expanded-bed height was not systematically recorded at each superficial linear velocity; therefore, bed expansion ratio, expanded-bed voidage, and minimum fluidization or expansion velocity could not be determined. Consequently, the present assessment should be interpreted as a comparative evaluation of hydraulic stability among the tested configurations rather than as a complete fluidization characterization.

Following evaluation of the downflow configuration, upflow operation was investigated using the 90 mm column containing 470 or 705 g of β-CD-EPI. In this configuration, liquid was introduced through the bottom of the column, producing visually observable mobilization and expansion of the hydrated polymer bed during operation. Downflow and upflow configurations were subsequently compared at equivalent adsorbent loadings and superficial linear velocities to determine the operating mode providing the greatest hydraulic stability and the lowest tendency toward bed compaction. The hydrodynamic conditions identified from these experiments were then used to define the operating configuration for the subsequent continuous-flow adsorption assays. The downflow screening experiments and the direct downflow–upflow comparison at a superficial linear velocity of 12 m h^−1^ were performed in triplicate (n = 3), and the corresponding pressure-drop data are expressed as mean ± standard deviation (SD). The broader upflow velocity screening presented in Figure S2 was conducted as an operational screening test and is reported descriptively.

### 2.5. Continuous-Flow Adsorption Experiments

Following the comparative hydrodynamic evaluation, continuous-flow adsorption experiments were performed using the 90 mm nominal-diameter column operated in upflow mobile-bed mode, with visually observed bed expansion and particle movement. The column was loaded with 705 g of β-CD-EPI polymer, corresponding to an approximate hydrated adsorbent-bed volume (V_ads_) of 3 L and a bed depth of approximately 0.54 m. The polymer was supported on a layer of activated silica glass covering the lower retaining element of the column. Feed solutions were introduced through the bottom of the column and passed upward through the hydrated adsorbent bed before being collected in the outlet vessel.

Three nominal contact times ($t_{C}$) of 3, 5, and 10 min were evaluated. The corresponding volumetric flow rate was calculated from the hydrated adsorbent-bed volume according to Equation (1):

(1) $$t_{C}=\frac{V_{ads}}{Q}$$

where t_C_ is the nominal contact time, V_ads_ is the hydrated adsorbent bed volume and Q is the volumetric flow rate. The resulting flow rates, superficial linear velocities and hydraulic loadings corresponding to each nominal contact time are summarized in Table 1.

Because nominal contact time was adjusted by changing the volumetric flow rate, each contact-time condition was intrinsically associated with a different superficial linear velocity. Therefore, the effects of nominal residence time and superficial linear velocity were not independently evaluated. The 3, 5, and 10 min conditions should consequently be interpreted as coupled hydraulic operating conditions used to compare prototype performance within the hydrodynamically stable range identified during the preliminary column assessment.

#### 2.5.1. Single-Contaminant Experiments

Single-solute continuous-flow experiments were performed independently for the five target contaminants: furosemide, hydrochlorothiazide, acetaminophen, ciprofloxacin, and cyproconazole. Separate feed solutions were prepared in tap water at initial concentrations (C_0_) of 5 mg L^−1^ for furosemide, 5 mg L^−1^ for hydrochlorothiazide, 10 mg L^−1^ for acetaminophen, 9.6 mg L^−1^ for ciprofloxacin, and 7 mg L^−1^ for cyproconazole. These concentrations were selected as controlled high-loading challenge conditions to enable comparative assessment of contaminant-specific adsorption during the short-duration laboratory experiments. They were not intended to reproduce the trace concentrations typically occurring in municipal wastewater, and the resulting removal efficiencies should not be interpreted as direct predictors of performance at environmentally relevant concentrations. Each contaminant was evaluated under the three coupled hydraulic operating conditions described in Section 2.5, corresponding to nominal contact time/superficial linear velocity combinations of 3 min/10.7 m h^−1^, 5 min/6.4 m h^−1^, and 10 min/3.2 m h^−1^. Samples were collected from the influent and column effluent for each operating condition and analyzed as described in Section 2.7. The resulting inlet and outlet concentrations were used to determine the normalized effluent concentration (C/C_0_) and removal efficiency for each compound.

#### 2.5.2. Multicomponent Adsorption Experiments

Competitive adsorption under continuous-flow conditions was evaluated using binary and ternary contaminant mixtures. For the ternary experiment, the feed solution contained cyproconazole (7 mg L^−1^), acetaminophen (10 mg L^−1^), and ciprofloxacin (9.6 mg L^−1^). The mixture was processed through the β-CD-EPI polymer bed under the three coupled hydraulic operating conditions described in Section 2.5: 3 min/10.7 m h^−1^, 5 min/6.4 m h^−1^, and 10 min/3.2 m h^−1^. Influent and effluent samples were collected for each condition, and the concentration of each contaminant was determined independently as described in Section 2.7. Compound-specific C/C_0_ values and removal efficiencies were then calculated to assess the influence of simultaneous exposure on contaminant retention.

A second multicomponent experiment was subsequently performed using a binary mixture containing cyproconazole (7 mg L^−1^) and acetaminophen (10 mg L^−1^). The binary system was evaluated under the 3 min/10.7 m h^−1^ and 5 min/6.4 m h^−1^ coupled hydraulic conditions using the same continuous-flow configuration. Influent and effluent concentrations were determined separately for both compounds and compared with those obtained in the corresponding single-solute experiments.

#### 2.5.3. Wastewater-Matrix Experiments

The influence of the wastewater matrix on contaminant removal was evaluated using secondary-treated effluent collected from the Cabezo Beaza municipal wastewater treatment plant (Cartagena, southeastern Spain). Matrix characterization was carried out in accordance with Royal Decree 1085/2024 [22]. The main physicochemical characteristics of the secondary-treated effluent used in the matrix experiment are summarized in Table 2.

For the continuous-flow matrix experiment, 50 L of this secondary-treated effluent was spiked with cyproconazole to obtain an initial concentration (C_0_) of 7 mg L^−1^. The fortified wastewater was processed through the β-CD-EPI polymer bed at nominal contact times of 3, 5, and 10 min using the same column configuration and hydraulic conditions applied in the corresponding single-solute experiment in tap water. Influent and effluent samples were collected at each operating condition and analyzed as described in Section 2.7. Compound-specific C/C_0_ values and removal efficiencies were subsequently compared with those obtained in tap water (pH 8.3, electrical conductivity of 1200 µS cm^−1^, total hardness of 280 mg L^−1^ CaCO_3_, 0.8 mg L^−1^ free chlorine, 50 mg L^−1^ Ca^2+^, 6 mg L^−1^ NO_3_^−^) at the same cyproconazole concentration to assess the effect of wastewater constituents on adsorption performance.

### 2.6. Desorption and Post-Desorption Rinsing

#### 2.6.1. Continuous-Flow Desorption Experiments

The desorption step required for regeneration of the β-CD-EPI polymer was evaluated in a separate set of continuous-flow experiments using the 63 mm nominal-diameter column. The column was loaded with 100 g of β-CD-EPI polymer supported on a layer of activated silica glass covering the lower retaining element. Cyproconazole was selected as the model contaminant. Prior to desorption, the adsorbent was loaded by passing a 10 mg L^−1^ cyproconazole solution through the column at a flow rate of 4.8 L h^−1^, corresponding to a nominal contact time of 5 min. The cyproconazole concentration in the column effluent was determined to estimate the amount retained by the polymer before desorption.

Desorption was subsequently performed using 220 mM acetate buffer adjusted to pH 4.0 at a desorbent-to-adsorbent ratio of 10 mL g^−1^. The regenerating solution was introduced through the desorption line and passed through the polymer bed in downflow mode. Independent desorption experiments were initially conducted at nominal contact times of 5 and 10 min. An additional experiment at 15 min was subsequently included to determine whether extending the contact time further increased cyproconazole recovery. The 5, 10, and 15 min desorption conditions were operated at flow rates of 4.8, 2.4, and 1.6 L h^−1^, corresponding to superficial linear velocities of 1.88, 0.94, and 0.63 m h^−1^, respectively. The desorption eluate was collected downstream of the column and analyzed for recovered cyproconazole as described in Section 2.7.

#### 2.6.2. Post-Desorption Rinsing

Following each desorption experiment, the polymer bed was rinsed with demineralized water to displace the residual pH 4.0 acetate buffer while maintaining the polymer in its hydrated state. The rinsing procedure was evaluated in two successive stages. First, low-flow rinsing rates of 2, 3, 4, and 5 BV h^−1^ were compared over 30 min. Effluent pH was monitored throughout the experiment and used as the quantitative process-control variable. The practical rinsing endpoint was defined as recovery of the effluent to the near-neutral operating range observed for the incoming rinse water, approximately pH 6.8–7.2 under the experimental conditions.

At 2 BV h^−1^, the effluent pH increased progressively from approximately 4.3 to 6.3 after 30 min, whereas final values of approximately 7.1–7.2 were obtained at 3–5 BV h^−1^. A subsequent 10 min high-flow rinsing stage was then compared at 10, 20, 30, and 40 BV h^−1^. Starting from an effluent pH of approximately 6.3, the corresponding final values were approximately 6.8, 6.9, 7.0, and 7.0, respectively. Because increasing the high-flow rinsing rate above 10 BV h^−1^ produced only a limited additional pH increase, the lowest tested rate was retained for this second stage.

On this basis, the preliminary operating sequence selected for the tested configuration consisted of a 30 min low-flow rinse at 2 BV h^−1^ (6 L h^−1^), followed by a 10 min high-flow rinse at 10 BV h^−1^ (30 L h^−1^). These stages corresponded to approximately 1.0 and 1.67 bed volumes of rinse water, respectively, giving a total rinsing demand of approximately 2.67 BV. The sequence was selected as an operational compromise that combined gradual buffer displacement at low hydraulic loading with a short higher-flow polishing step, while restoring the effluent to the near-neutral operating range.

Because these rinsing experiments were intended to establish operational flow-rate and time requirements for restoring the column effluent to the target pH, the recorded pH profiles were treated as process-control data rather than as datasets for statistical comparison.

### 2.7. Analytical Determination and Data Treatment

All influent and effluent samples generated during the continuous-flow adsorption, multicomponent, wastewater-matrix, and desorption experiments were analyzed by ultra-high-performance liquid chromatography coupled to high-resolution tandem mass spectrometry (UHPLC-HRMS/MS) (Redwood City, CA, USA). The analytical procedure was based on a previously validated and published methodology developed by our group using the same X500R QTOF platform [10] and was adapted to the five target analytes investigated here. In the present study, this established chromatographic methodology was used as an analytical tool to quantify changes between influent and effluent concentrations under the controlled high-loading conditions applied; analytical-method development and trace-level environmental monitoring were not objectives of the study.

Analyses were performed using an X500R quadrupole time-of-flight (QTOF) high-resolution mass spectrometer (SCIEX, Redwood City, CA, USA) equipped with a Turbo V™ ion source and coupled to a UHPLC system controlled by SCIEX OS software (version 2.0, SCIEX, Redwood City, CA, USA). Chromatographic separation was achieved using an end-capped Purospher STAR RP-18 column (100 × 2 mm, 2 µm particle size; Merck, Darmstadt, Germany) maintained at 40 °C. The injection volume was 10 µL, and the mobile-phase flow rate was 0.4 mL min^−1^.

Separate acquisitions were performed under positive and negative electrospray ionization conditions. In positive-ion mode, the aqueous mobile phase consisted of water containing 0.1% (*v*/*v*) formic acid and 5 mM ammonium acetate, while acetonitrile was used as the organic phase. The initial acetonitrile content (5%) was maintained for 0.5 min and then increased to 98% over 15.5 min. The 98% organic composition was maintained for 3 min before returning to the initial conditions, followed by a 5 min column re-equilibration period. The total chromatographic analysis time was approximately 24 min.

For negative-ion acquisition, the mobile phases consisted of water containing 5 mM ammonium acetate and acetonitrile. The initial acetonitrile content (5%) was maintained for 0.5 min, increased to 73% over 11.5 min, and subsequently increased to 100% over 0.5 min. The 100% organic condition was maintained for 40 s before returning to the initial composition, followed by a 1.8 min column re-equilibration period. The total chromatographic analysis time was 15 min.

Acetaminophen, ciprofloxacin, and cyproconazole were monitored under positive-ion conditions, whereas furosemide and hydrochlorothiazide were determined under negative-ion conditions. High-resolution MS/MS data were acquired in multiple-reaction-monitoring high-resolution (MRMHR) mode. The MS acquisition range was *m*/*z* 100–800, and MS/MS spectra were recorded over the *m*/*z* range of 50–700 using an accumulation time of 25 ms. For each target analyte, the precursor ion and two characteristic product ions were monitored, with one product-ion transition used for quantitative determination and the second for confirmatory identification. The compound-specific precursor ions and corresponding quantifier and qualifier product ions used for MRMHR acquisition are summarized in Table S1 (Supplementary Materials).

Quantification was performed using analyte-specific external calibration curves prepared from mixed standard solutions. A linear calibration range of 5–1000 µg L^−1^ was used for all five target analytes. Experimental samples were diluted as necessary to ensure that analyte responses remained within this calibration interval. In multicomponent samples, chromatographic separation combined with accurate-mass precursor- and product-ion monitoring allowed each target compound to be selectively identified and quantified independently under the analytical conditions applied.

For the wastewater-matrix experiments, cyproconazole was intentionally added to the secondary-treated effluent at a concentration of 7 mg L^−1^ to evaluate adsorption performance under a controlled high-loading condition in a real wastewater matrix rather than to determine environmentally occurring trace concentrations; therefore, no analyte preconcentration step was required. Prior to UHPLC-HRMS/MS analysis, influent and effluent samples were filtered through disposable 0.45 µm hydrophilic polyethersulfone (PES) syringe filters to remove suspended particulate matter and were diluted as necessary to bring cyproconazole responses within the 5–1000 µg L^−1^ working calibration range. The combined filtration and dilution procedure removed suspended material and reduced the amount of wastewater-matrix constituents introduced into the electrospray ionization source. Cyproconazole was quantified using the same chromatographic and high-resolution mass-spectrometric criteria described above.

For the wastewater-matrix experiment, the analytical comparison was based on paired influent and effluent samples collected from the same fortified wastewater batch and subjected to identical filtration, dilution, and instrumental procedures. Because cyproconazole was added at 7 mg L^−1^ and the samples were diluted to the linear calibration range, no preconcentration was required and analytical sensitivity was not a limiting factor. This fit-for-purpose approach was used to determine relative changes in cyproconazole concentration and calculate C/C_0_ under the tested high-loading conditions. A complete trace-level environmental-method validation was outside the analytical scope of the present adsorption study.

Continuous-flow adsorption performance was expressed as the normalized effluent concentration (*C*/*C*_0_), where *C*_0_ and *C* represent the experimentally measured influent and corresponding effluent concentrations, respectively. Removal efficiency was calculated according to Equation (2):

(2) $$Removal\;efficiency(\%)=\left(\frac{C_{0}-C}{C_{0}}\right)\times 100$$

Both *C*/*C*_0_ and removal efficiency were calculated independently for each target contaminant and used to compare adsorption performance as a function of contaminant identity, nominal contact time, mixture composition, and aqueous matrix. For the wastewater-matrix experiment, the paired measurement of influent and effluent concentrations under each operating condition allowed the removal response to be calculated relative to the corresponding influent concentration in the same fortified wastewater batch.

For the desorption experiments, cyproconazole concentrations in the loading and desorption fractions were determined using the same UHPLC-HRMS/MS procedure. Desorption efficiency was calculated as the fraction of the contaminant mass previously retained by the polymer that was subsequently recovered in the acetate-buffer eluate according to Equation (3):

(3) $$Desorption\;efficiency(\%)=\left(\frac{m_{des}}{m_{ads}}\right)\times 100$$

where *m_des_* is the mass of cyproconazole recovered during desorption and *m_ads_* is the mass retained by the β-CD-EPI polymer bed during the preceding adsorption step. Both masses were calculated from the measured cyproconazole concentrations and the corresponding solution volumes. Post-desorption rinsing was monitored by measuring the pH of the column effluent, and the rinsing sequence was considered complete when the outlet pH approached that of the incoming rinse water.

Unless otherwise stated, adsorption experiments used for quantitative removal comparisons were performed in triplicate (n = 3), and results are expressed as mean ± SD. Desorption and post-desorption rinsing tests were conducted as operational optimization assays to define process set points and endpoint conditions; therefore, the corresponding recovery efficiencies and effluent-pH profiles are reported as operational values without dispersion statistics.

## 3. Results and Discussion

### 3.1. Hydrodynamic Behavior and Selection of the Operating Mode

Hydrodynamic characterization showed that the operating configuration was a critical determinant of the feasibility of continuous treatment with the β-CD-EPI polymer. Under downflow conditions, the hydrated polymer bed exhibited a pronounced tendency toward compaction, and the resulting pressure drop increased with both superficial linear velocity and polymer loading. In the 63 mm column containing 235 g of polymer, the pressure drop remained moderate at superficial velocities below 12 m h^−1^, whereas operation at 20 m h^−1^ resulted in rapid pressure development and marked bed compaction within approximately 5 min. Increasing the polymer loading further intensified this behavior. With 705 g of β-CD–EPI, a pressure drop close to 1 bar was reached after approximately 10 min even at 4 m h^−1^, while at 12 m h^−1^, rapid pressure development resulted in termination of stable operation after approximately 5 min.

Increasing the nominal column diameter to 90 mm partially improved the hydraulic behavior but did not eliminate the tendency of the hydrated β-CD-EPI bed to compact under downflow operation. At the lowest polymer loading (235 g), the larger effective cross-sectional area allowed for comparatively stable short-term operation, with the pressure drop remaining below approximately 0.3 bar at superficial velocities up to 16 m h^−1^ and below 0.5 bar at 20 m h^−1^ during the initial operating period. However, when operation at the highest velocity was extended to 20 min, the pressure drop increased to approximately 1.5 bar, demonstrating that the larger column delayed rather than prevented bed compaction. This limitation became more pronounced as polymer loading increased: with 470 and 705 g of β-CD–EPI, rapid pressure development and bed compaction were again observed within the 8–12 m h^−1^ range, resulting in operating periods of only a few minutes before excessive hydraulic resistance developed. Thus, increasing column diameter reduced the initial resistance to liquid flow but was insufficient, by itself, to provide stable continuous operation in the downflow configuration. The complete hydrodynamic screening under downflow operation, including the effects of column diameter, polymer loading, and superficial linear velocity, is presented in Figure S1.

**Figure 1:**
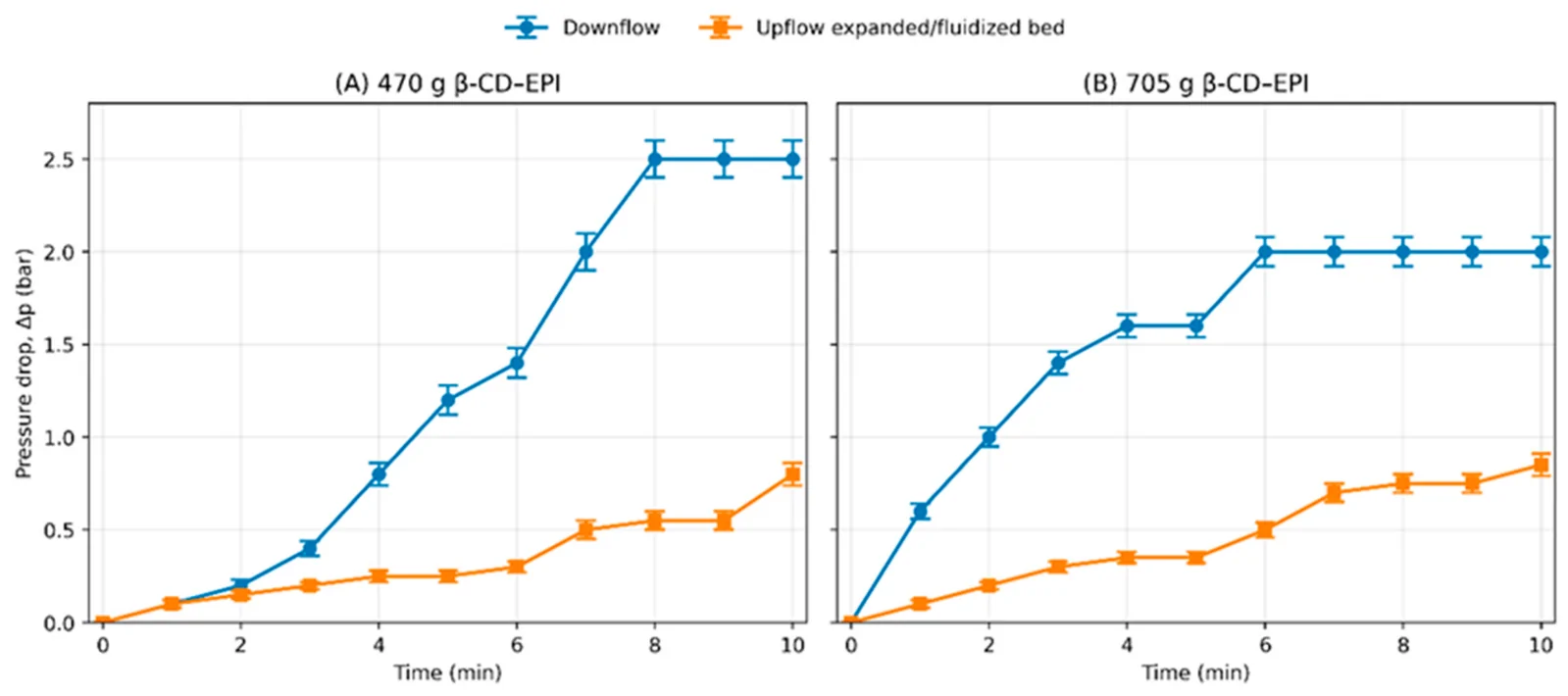
Effect of flow direction on the hydrodynamic behavior of the β-CD-EPI polymer bed in the 90 mm column at a superficial linear velocity of 12 m h^−1^. Pressure-drop evolution under downflow and upflow mobile-bed operation using (**A**) 470 g and (**B**) 705 g of β-CD-EPI polymer; bed expansion and particle movement under upflow conditions were assessed visually. Data are presented as mean ± SD (n = 3).

A markedly different hydraulic response was observed when the 90 mm column was operated in upflow mobile-bed mode. With both 470 and 705 g of β-CD-EPI polymer, superficial linear velocities below approximately 12 m h^−1^ produced low and comparatively stable pressure drops, while visual inspection showed particle movement and expansion of the hydrated bed within the central region of the column. Stable operation could also be maintained at velocities up to 16 m h^−1^ for short operating periods. At 20 m h^−1^, however, the pressure drop progressively increased, mainly in association with polymer-particle entrainment toward the upper retaining element during prolonged operation. The complete pressure-drop profiles obtained under upflow mobile-bed conditions are shown in Figure S2. Direct comparison of downflow and upflow configurations at equivalent polymer loadings and a superficial linear velocity of 12 m h^−1^ confirmed that upflow operation substantially reduced pressure development and the tendency of the hydrated bed to compact (Figure 1).

Because bed height was not quantitatively recorded as a function of superficial linear velocity, the visual observations cannot be used to determine an expansion ratio, expanded-bed voidage, or minimum fluidization or expansion velocity. The hydrodynamic comparison is therefore based quantitatively on pressure-drop behavior and operational stability, with the observed particle mobilization and bed expansion providing qualitative supporting evidence.

The contrasting behavior observed between downflow and upflow operation can be related to the swelling and deformability of the hydrated β-CD-EPI polymer network. Under downflow conditions, the hydraulic force acts in the same direction as gravitational settling, progressively compressing the polymer bed, reducing interparticle void space, and increasing resistance to liquid passage. Once compacted, the hydrated polymer formed cohesive regions that were difficult to redisperse, even after water backwashing, which explains the rapid recovery of high pressure drop when downflow operation was resumed. In contrast, upward flow counteracted gravitational settling and maintained the polymer particles in a dynamically expanded state, thereby preserving interparticle void space and limiting the progressive increase in hydraulic resistance. This interpretation is consistent with the general behavior of upflow adsorption systems in which particle mobilization and increased interparticle spacing can reduce excessive pressure development and channeling compared with compacted downflow beds [16]. However, because expanded-bed height, voidage, and minimum fluidization or expansion velocity were not determined, the present results do not establish a formally characterized fluidized-bed regime. Moreover, the feasibility of operating CD-based polymer adsorbents under fluidized conditions has previously been demonstrated at pilot scale for the removal of micropollutants from treated municipal wastewater [17].

Based on this comparative hydrodynamic evaluation, the 90 mm column operated in upflow mobile-bed mode was selected for the subsequent adsorption experiments. A polymer loading of 705 g, corresponding to an approximately 3 L hydrated bed and a bed depth of about 0.54 m, was retained as the working configuration because it provided sufficient adsorbent volume while remaining hydraulically stable under the selected operating conditions. A superficial linear velocity of approximately 12 m h^−1^ was therefore adopted as a conservative, configuration-specific practical upper operating boundary for the subsequent adsorption experiments. This value was based on pressure-drop stability and the absence of excessive particle accumulation at the upper retaining element under the tested conditions and should not be interpreted as a minimum fluidization velocity. Accordingly, the nominal contact times of 3, 5, and 10 min used in the adsorption experiments corresponded to superficial linear velocities of 10.7, 6.4, and 3.2 m h^−1^, respectively, all below the conservative practical operating boundary identified during the comparative hydrodynamic evaluation. Selecting this operating range ensured that all subsequent adsorption experiments were performed under conditions that had shown comparatively stable pressure-drop behavior. However, because each nominal contact time was associated with a different superficial linear velocity, the adsorption results represent comparisons among coupled hydraulic operating conditions and cannot be interpreted as the independent effect of residence time.

### 3.2. Contaminant-Specific Adsorption Under Continuous-Flow Conditions

**Figure 2:**
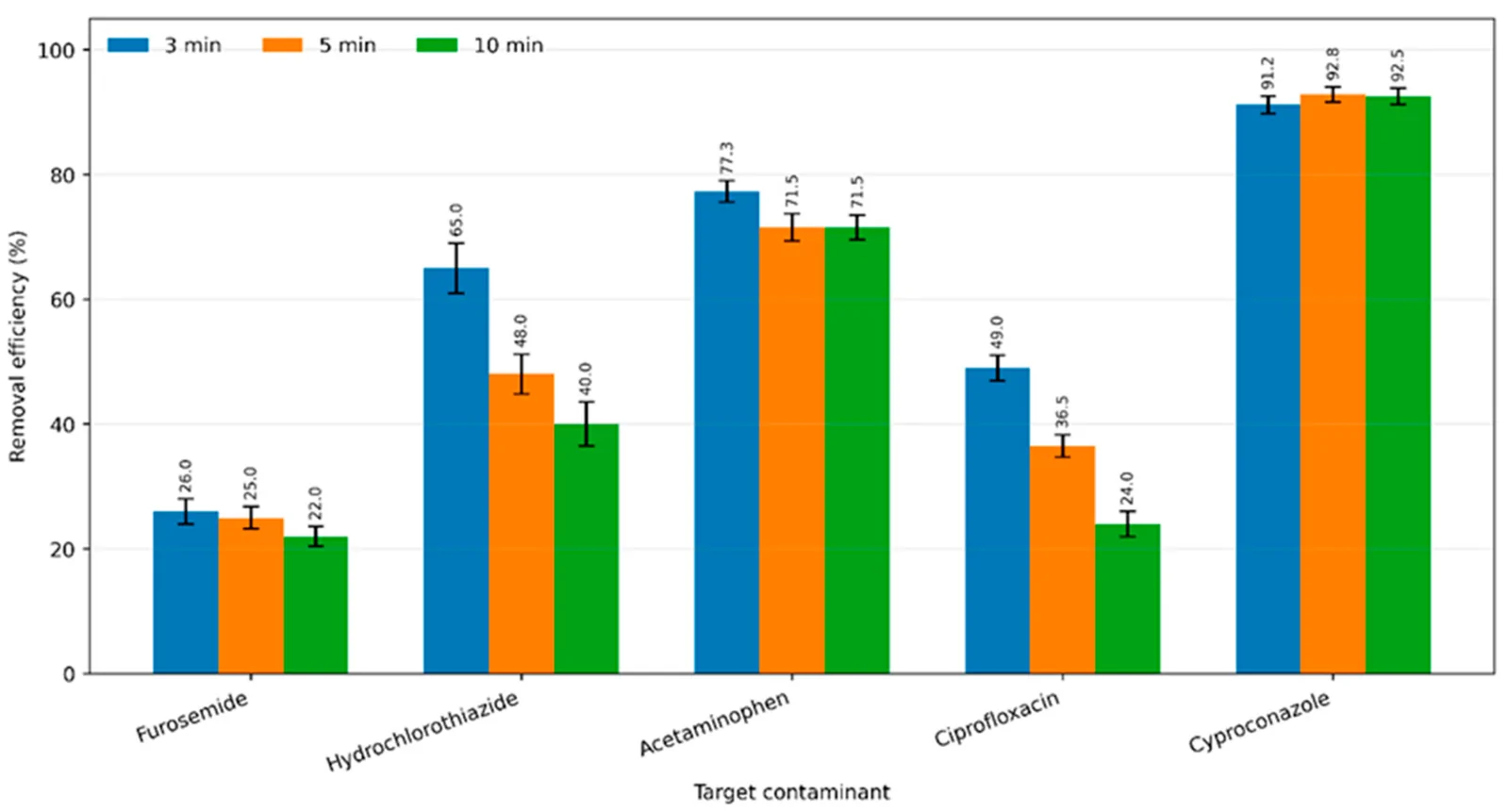
Continuous-flow removal efficiencies of furosemide, hydrochlorothiazide, acetaminophen, ciprofloxacin, and cyproconazole under three coupled hydraulic operating conditions: 3 min/10.7 m h^−1^, 5 min/6.4 m h^−1^, and 10 min/3.2 m h^−1^, where the first value indicates nominal contact time and the second indicates superficial linear velocity. Initial contaminant concentrations were 5 mg L^−1^ for furosemide and hydrochlorothiazide, 10 mg L^−1^ for acetaminophen, 9.6 mg L^−1^ for ciprofloxacin, and 7 mg L^−1^ for cyproconazole. Data are presented as mean ± SD (n = 3).

Once a hydraulically stable operating configuration had been established, the adsorption performance of the β-CD-EPI polymer was evaluated for each of the five target contaminants. A pronounced compound-dependent response was observed under continuous-flow conditions. Cyproconazole showed the highest retention, with removal efficiencies above 90% at all three nominal contact times, whereas acetaminophen also exhibited consistently high removal, reaching 77.3% at 3 min and remaining at approximately 72% at 5 and 10 min. Hydrochlorothiazide displayed an intermediate response, with removal decreasing from more than 65% at 3 min to approximately 48% and 40% at 5 and 10 min, respectively. Ciprofloxacin showed lower retention, decreasing from approximately 50% at 3 min to 36% at 5 min and 24% at 10 min, while furosemide was the least effectively retained compound, with removal remaining below 30% throughout the investigated operating range. Thus, under the same reactor configuration, although at compound-specific influent concentrations, the β-CD-EPI polymer exhibited markedly different adsorption responses depending on contaminant identity. The corresponding removal efficiencies are compared in Figure 2.

Notably, the operating condition corresponding to a nominal contact time of 3 min produced equal or higher apparent removal than the 5- and 10-min conditions for most of the contaminants evaluated. This pattern should not be interpreted as evidence that reducing residence time independently improved adsorption. In the present configuration, nominal contact time was modified by changing the volumetric flow rate; therefore, the 3-, 5-, and 10-min conditions were associated with superficial linear velocities of 10.7, 6.4, and 3.2 m h^−1^, respectively. Each condition consequently represented a distinct combination of nominal residence time, superficial linear velocity, solid–liquid contact, and mass-transfer environment rather than an isolated variation in contact time.

The present experimental design does not allow the individual contribution of these factors to be resolved. In particular, although changes in upward velocity may affect particle mobility, bed expansion, boundary-layer resistance, and external mass transfer, these effects were not independently quantified during the adsorption experiments. The higher removal observed under the 3 min/10.7 m h^−1^ condition should therefore be interpreted as an apparent response to that coupled hydraulic operating condition and not as a direct consequence of the shorter residence time or higher superficial linear velocity alone. Cyproconazole was comparatively insensitive to the three tested conditions, maintaining removal above 90% throughout the investigated range.

These results emphasize that nominal residence time should not be interpreted independently of the associated hydrodynamic regime when evaluating adsorption in an upflow hydrated-polymer bed. Experiments in which residence time and superficial linear velocity are independently varied, for example, by modifying bed volume or column geometry while maintaining one of these variables constant, will be required to determine their respective contributions to contaminant removal.

The comparison between furosemide and hydrochlorothiazide was particularly informative because these two compounds had been used in the preceding batch study to derive the adsorption parameters employed in prototype design [20]. Under batch conditions, furosemide showed a higher Langmuir-derived maximum adsorption capacity than hydrochlorothiazide (1.282 versus 0.844 mg g^−1^) and reached apparent equilibrium considerably faster, within approximately 10–20 min compared with 40–60 min for hydrochlorothiazide. Nevertheless, their relative behavior was reversed under continuous-flow operation. At the same influent concentration of 5 mg L^−1^, furosemide removal remained below 30% under all three coupled hydraulic conditions, whereas hydrochlorothiazide removal exceeded 65% under the 3 min/10.7 m h^−1^ condition and was approximately 48% and 40% under the 5 min/6.4 m h^−1^ and 10 min/3.2 m h^−1^ conditions, respectively. Thus, neither the higher batch-derived adsorption capacity nor the shorter batch equilibration time of furosemide translated into superior removal under dynamic operation. This divergence provides direct experimental evidence that batch-derived equilibrium and kinetic parameters are valuable inputs for preliminary process design but cannot, by themselves, predict compound-specific removal in a continuously operated β-CD-EPI polymer bed.

Acetaminophen and ciprofloxacin further illustrated the strong dependence of continuous-flow adsorption on contaminant identity. At comparable influent concentrations of 10 and 9.6 mg L^−1^, acetaminophen was consistently well retained, with removal efficiencies of 77.3% at 3 min and approximately 72% at both 5 and 10 min, corresponding to C/C_0_ values of approximately 0.23–0.28. In contrast, ciprofloxacin removal was approximately 50% at 3 min and decreased to 36% and 24% at 5 and 10 min, respectively, with the corresponding C/C_0_ values increasing from approximately 0.51 to 0.76. These markedly different contaminant responses under the same set of coupled hydraulic conditions demonstrate that neither nominal residence time nor superficial linear velocity alone can account for the observed adsorption performance. The results are also consistent with contaminant-specific differences in apparent affinity for the β-CD-EPI network, although the individual contributions of hydraulic and molecular factors cannot be resolved from the present experimental design.

Previous studies with EPI-cross-linked cyclodextrin polymers have likewise shown that pollutant retention cannot generally be explained by a single physicochemical descriptor, but results from the combined influence of host–guest inclusion, hydrophobic and hydrophilic interactions, hydrogen bonding, molecular size, stereochemistry, and the structural characteristics of the cross-linked polymer network [8,10,12,13]. The different adsorption behavior observed here is therefore consistent with the multifunctional nature of β-CD-EPI materials, in which both the cyclodextrin cavities and the surrounding polymer matrix may contribute to solute retention. However, because the individual contribution of these interactions was not experimentally resolved in the present study, the results should be interpreted as evidence of contaminant-specific apparent affinity under dynamic conditions rather than as proof of a particular molecular adsorption mechanism.

Additional mechanistic context is provided by previous experimental and computational studies of β-CD-EPI polymer systems. In the preceding batch investigation, the equilibrium data for furosemide and hydrochlorothiazide fitted the Freundlich model more closely than the Langmuir model, with R^2^ values of 0.991 and 0.905 for Freundlich compared with 0.516 and 0.514 for Langmuir, respectively [20]. This behavior is consistent with adsorption within a heterogeneous polymer network containing interaction domains with different apparent affinities rather than with adsorption on a uniform population of equivalent sites. The multistage intraparticle-diffusion profiles obtained in that study also indicated that diffusion through the hydrated polymer network contributed to adsorption but was not the sole rate-controlling process [20]. In a separate investigation using a related commercial β-CD-EPI bead polymer and a structurally diverse group of pharmaceuticals, experimental retention was evaluated together with molecular-docking calculations [10]. The computed binding energies for association with β-CD provided a better qualitative explanation of compound-dependent retention than water solubility alone, for which only a weak correlation with removal was obtained (R^2^ = 0.30) [10]. Taken together, these previous findings support a combined adsorption framework involving β-CD host–guest association, interactions with heterogeneous domains of the EPI-cross-linked network, and diffusion through the hydrated polymer. Nevertheless, model fitting and molecular docking provide indirect mechanistic evidence and do not quantify the individual contribution of these processes under the present continuous-flow conditions.

Cyproconazole exhibited the most favorable continuous-flow response among the five contaminants evaluated. At an influent concentration of 7 mg L^−1^, removal remained above 90% at nominal contact times of 3, 5, and 10 min, corresponding to C/C_0_ values below approximately 0.1. Unlike the other compounds, its retention was therefore comparatively insensitive to the changes in superficial linear velocity associated with the three operating conditions. Such behavior is consistent with a strong apparent interaction with the β-CD-EPI adsorbent and with the ability of CD-based polymeric networks to retain organic compounds through the combined contribution of host–guest inclusion and interactions with the cross-linked polymer matrix [8,10]. Although the relative contribution of these mechanisms was not directly determined, the consistently high removal of cyproconazole across the investigated hydrodynamic range indicates that its affinity for the adsorbent was sufficient to minimize the influence of the operating changes that affected the other contaminants. This behavior also supported its selection as the model compound for the subsequent evaluation of competitive adsorption, wastewater-matrix effects, and desorption.

Taken together, the single-contaminant experiments demonstrate that the transfer of batch-derived design parameters to continuous-flow adsorption is inherently compound-dependent. The β-CD-EPI polymer provided high removal for cyproconazole and acetaminophen, intermediate performance for hydrochlorothiazide, and substantially lower retention of ciprofloxacin and furosemide. Importantly, this ranking could not be predicted solely from the batch-derived adsorption capacities or equilibration times obtained during prototype design, as illustrated most clearly by the contrasting behavior of furosemide and hydrochlorothiazide. Batch studies therefore remain essential for initial characterization of polymer–solute interactions and for preliminary process design, but dynamic validation is required to account for the additional influence of hydrodynamics, mass-transfer conditions, and contaminant-specific affinity under continuous operation. The present results thus support the feasibility of continuous β-CD-EPI adsorption while defining an important limitation of direct batch-to-flow extrapolation: adsorption parameters obtained under equilibrium conditions should be regarded as design inputs rather than direct predictors of continuous-flow removal efficiency.

### 3.3. Competitive Adsorption in Multicomponent Systems

The presence of multiple contaminants markedly altered the adsorption response of the β-CD-EPI polymer. In the ternary system containing cyproconazole, acetaminophen, and ciprofloxacin, removal efficiencies were lower than those obtained in the corresponding single-contaminant experiments, although the magnitude of the decrease was strongly compound-dependent. Cyproconazole remained the most effectively retained compound, with removal efficiencies ranging from approximately 48% to 74% across the three operating conditions, compared with >90% when evaluated individually. The effect of the mixture was more pronounced for acetaminophen, whose removal decreased from approximately 72–77% in single-solute experiments to approximately 33–35% in the ternary system. Ciprofloxacin was also strongly affected, with removal efficiencies of approximately 18–20%, compared with approximately 24–50% when evaluated individually. Thus, although the relative retention order cyproconazole > acetaminophen > ciprofloxacin was preserved, simultaneous exposure substantially reduced the removal efficiency of all three contaminants.

The reduction in removal observed in the ternary system is consistent with competitive interactions within the heterogeneous β-CD-EPI network. Because the polymer provides both cyclodextrin cavities and interaction domains within the EPI-cross-linked matrix, simultaneously present solutes may compete for partially overlapping adsorption environments rather than for a single uniform class of sites. Previous studies with cyclodextrin–EPI polymers have shown that contaminant retention can involve the combined contribution of host–guest inclusion, hydrophobic and hydrophilic interactions, hydrogen bonding, molecular dimensions, stereochemistry, and the structural characteristics of the polymer network [8,10,12,13]. Competition within such a multifunctional material is therefore expected to be contaminant-specific.

The different sensitivity of the three contaminants to multicomponent adsorption is also consistent with their contrasting molecular structures and ionization behavior under the experimental conditions. At the tap-water pH used in these experiments (pH 8.3), cyproconazole is expected to occur predominantly in its neutral form because of the weak basic character of its triazole functionality [23]. Its comparatively hydrophobic character, together with aromatic and chlorinated structural domains, may favor association with the hydrophobic β-CD cavities and less polar regions of the cross-linked network, while its hydroxyl and triazole groups provide additional potential interaction sites. Acetaminophen is also expected to remain predominantly neutral at this pH but is considerably more hydrophilic; its phenolic hydroxyl and amide functionalities may participate in hydrogen-bonding interactions with the hydroxyl- and ether-rich β-CD-EPI network, whereas its lower hydrophobicity may provide a weaker driving force for cavity-associated interactions. In contrast, ciprofloxacin is an amphoteric compound with pKa values of approximately 6.1 and 8.7 and is therefore predominantly zwitterionic at pH 8.3, with an appreciable contribution from the anionic form [24]. Its ionized character, strong hydration, and greater structural complexity may reduce favorable association with hydrophobic β-CD domains and make its retention more sensitive to the presence of competing solutes. These structural and speciation differences are consistent with the experimentally preserved affinity order of cyproconazole > acetaminophen > ciprofloxacin, although the individual contribution of each interaction mechanism cannot be resolved from the present experiments alone.

The ability of cyproconazole to maintain the highest removal efficiency in the presence of two competing pharmaceuticals therefore indicates a stronger apparent affinity for the β-CD-EPI polymer under dynamic multicomponent conditions. Conversely, the larger relative losses observed for acetaminophen and particularly ciprofloxacin suggest that their retention was more sensitive to the simultaneous presence of other solutes. Importantly, these results demonstrate that adsorption performance derived from single-contaminant experiments may substantially overestimate removal when several organic contaminants coexist. Multicomponent effects under flow-through conditions have also been reported for a chemically distinct β-CD adsorbent, in which the presence and accumulation of higher-affinity contaminants altered the retention of more weakly interacting species [15].

**Figure 3:**
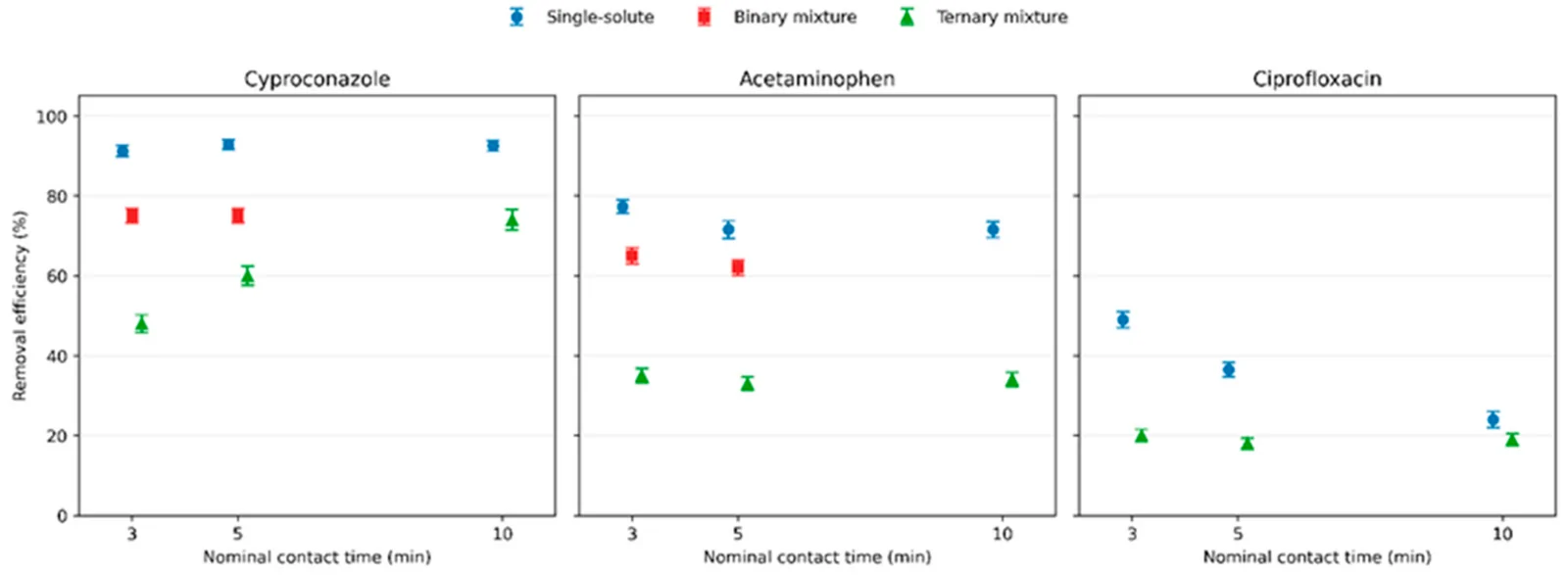
Effect of mixture composition on continuous-flow contaminant removal by the β-CD-EPI polymer. Removal efficiencies obtained under single-solute and ternary conditions are compared for cyproconazole, acetaminophen, and ciprofloxacin under the following coupled nominal contact time/superficial linear velocity conditions: 3 min/10.7 m h^−1^, 5 min/6.4 m h^−1^, and 10 min/3.2 m h^−1^. Binary experiments containing cyproconazole and acetaminophen were performed only under the 3 min/10.7 m h^−1^ and 5 min/6.4 m h^−1^ conditions; no binary experiment containing ciprofloxacin was conducted. Data are presented as mean ± SD (n = 3).

Reducing the mixture from three contaminants to the binary combination of cyproconazole and acetaminophen resulted in substantially higher removal efficiencies. Both compounds were removed at efficiencies above 50% under the 3 min/10.7 m h^−1^ and 5 min/6.4 m h^−1^ coupled hydraulic conditions. Cyproconazole maintained a removal efficiency of approximately 75%, corresponding to a C/C_0_ value of about 0.25, whereas acetaminophen removal ranged from approximately 62% to 65%, with C/C_0_ values of approximately 0.35–0.38. These values remained below those obtained in the corresponding single-contaminant experiments but were clearly higher than those measured in the ternary mixture. This partial recovery of adsorption performance when ciprofloxacin was absent from the feed is consistent with competitive effects among coexisting solutes and indicates that both mixture composition and combined solute loading can influence dynamic adsorption performance. Cyproconazole again showed the greatest resistance to multicomponent effects, whereas acetaminophen exhibited an intermediate response between its single-solute and ternary-mixture behavior. A direct comparison of the experimentally evaluated single-solute, binary, and ternary conditions is presented in Figure 3.

Taken together, the multicomponent experiments demonstrate that competitive adsorption is an important factor when extrapolating β-CD-EPI performance from simplified single-solute systems to more complex aqueous conditions. Although the polymer retained a clear preference for cyproconazole, the progressive decrease in removal from single-solute to multicomponent systems shows that continuous-flow adsorption depends not only on the apparent affinity of each contaminant but also on influent composition and the simultaneous presence of other solutes.

This effect was particularly evident for acetaminophen and ciprofloxacin, whose removal efficiencies were substantially reduced in the ternary mixture. Consequently, performance estimates based exclusively on isolated contaminants may overpredict treatment efficiency in complex waters.

Recent pilot- and full-scale adsorption studies using other adsorbent materials similarly demonstrate that competitive adsorption can limit the transferability of parameters derived from simplified experiments to realistic wastewater-treatment conditions [19]. From a process-design perspective, multicomponent effects should therefore be considered when defining polymer loading, operating conditions, and regeneration requirements for subsequent scale-up. These findings also provide the rationale for evaluating the β-CD-EPI system in an actual wastewater matrix, where dissolved organic matter, inorganic ions, and other constituents introduce an additional level of complexity.

### 3.4. Effect of the Wastewater Matrix on Adsorption Performance

**Figure 4:**
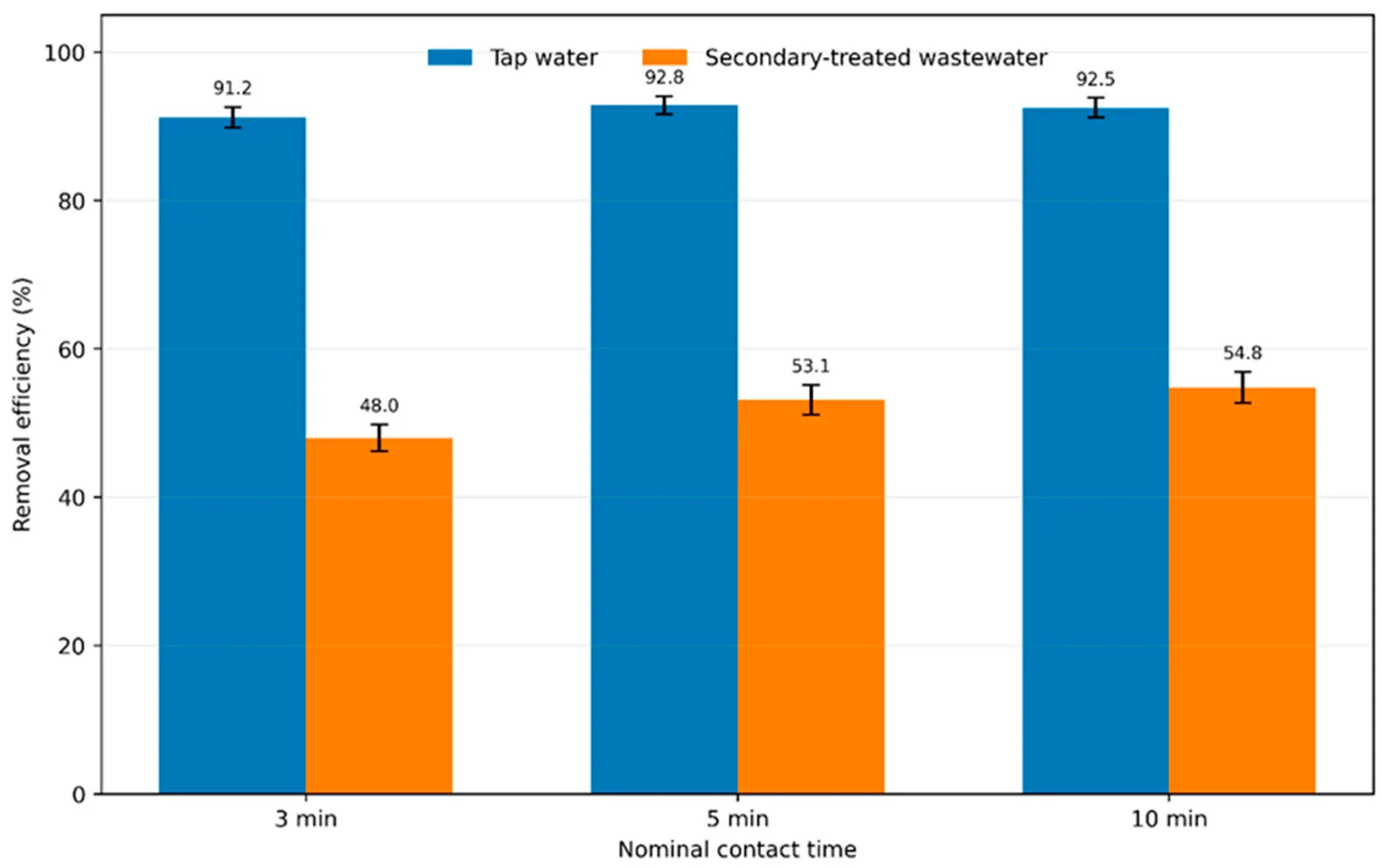
Effect of the aqueous matrix on cyproconazole removal by the β-CD-EPI polymer under continuous-flow conditions. Cyproconazole (*C*_0_ = 7 mg L^−1^) was evaluated in tap water and secondary-treated wastewater under three coupled nominal contact time/superficial linear velocity conditions: 3 min/10.7 m h^−1^, 5 min/6.4 m h^−1^, and 10 min/3.2 m h^−1^. Removal values were calculated from the experimentally measured paired influent and effluent concentrations. Data are presented as mean ± SD (n = 3).

The transition from tap water to secondary-treated wastewater produced a pronounced decrease in cyproconazole removal under continuous-flow conditions. When cyproconazole was evaluated individually in tap water at an influent concentration of 7 mg L^−1^, the β-CD-EPI polymer removed more than 90% of the contaminant across the three coupled hydraulic conditions. In contrast, when the same cyproconazole concentration was introduced into secondary-treated effluent from the Cabezo Beaza WWTP, removal efficiencies decreased to approximately 48–55%, corresponding to C/C_0_ values of approximately 0.52–0.45 for the 3 min/10.7 m h^−1^, 5 min/6.4 m h^−1^, and 10 min/3.2 m h^−1^ conditions. Thus, even though cyproconazole was the most strongly retained compound under controlled single-solute conditions, approximately half of the influent contaminant remained in the treated stream when the aqueous matrix was changed to secondary-treated wastewater. This marked reduction demonstrates a substantial effect of the aqueous matrix on cyproconazole removal by the β-CD-EPI polymer under the tested continuous-flow conditions. Because the individual contributions of the wastewater constituents were not isolated experimentally, the result should be interpreted as the overall effect of changing the aqueous matrix rather than as evidence of a single adsorption-inhibition mechanism. The comparison between tap water and secondary-treated wastewater is shown in Figure 4.

The lower apparent cyproconazole removal observed in secondary-treated wastewater is consistent with the combined influence of organic and inorganic constituents present in the effluent on the adsorption process. These matrix components may affect adsorption through several non-mutually exclusive processes, including competition for accessible interaction domains within the polymer, partial occupation of cyclodextrin cavities by dissolved organic molecules, and modification of solute–adsorbent interactions by the ionic composition of the aqueous phase.

Previous studies have shown that water-matrix composition can influence micropollutant adsorption by β-CD polymers, with dissolved organic matter and inorganic ions contributing to adsorption inhibition to different extents depending on the properties of both the adsorbent and the target compound [18]. Relatively small dissolved organic molecules may compete for access to CD cavities, while changes in ionic strength can modify electrostatic and other intermolecular interactions involved in contaminant retention [8,18]. However, because the individual contributions of the wastewater constituents were not isolated experimentally, the observed decrease in cyproconazole removal cannot be attributed to a single mechanism. Rather, the results demonstrate that adsorption performance measured in a simplified aqueous system was substantially modified when the β-CD-EPI polymer was exposed to a chemically more complex wastewater matrix.

Comparable matrix-related limitations have been reported for other adsorption systems. In activated-carbon adsorption, dissolved organic matter can reduce micropollutant uptake through a combination of pore blocking and competitive interactions, with the magnitude of this effect depending on the adsorbability of both the dissolved-organic-matter fractions and the target micropollutant [25,26]. Although these mechanisms cannot be directly extrapolated to the β-CD-EPI network, they support the broader conclusion that matrix effects are both adsorbent- and contaminant-dependent.

Importantly, sensitivity to the aqueous matrix is not uniform across β-CD-based adsorbents. In flow-through experiments performed with porous β-CD polymers and broad mixtures of micropollutants at environmentally relevant concentrations, adsorption was rapid but strongly compound-selective, while the influence of natural organic matter differed markedly from that observed for activated carbon [27]. These findings reinforce the need to establish matrix effects for each specific polymer–contaminant system rather than extrapolating behavior from the cyclodextrin scaffold alone. More recently, styrenic β-CD polymers evaluated directly in municipal wastewater showed compound-dependent removal and measurable dynamic adsorption behavior under rapid small-scale column operation [28], further demonstrating that the performance of cyclodextrin-based adsorbents in complex wastewater must be established experimentally under flow conditions.

From an operational perspective, the wastewater-matrix experiment defines an important boundary for extrapolating performance from controlled water to more realistic treatment conditions. Although the β-CD-EPI polymer retained a substantial fraction of cyproconazole in the secondary-treated effluent, the decrease from >90% removal in tap water to approximately 48–55% in wastewater demonstrates that performance obtained in simplified matrices cannot be applied directly to municipal effluents. Wastewater composition should therefore be incorporated explicitly into subsequent reactor development and scale-up, particularly when defining polymer loading, hydraulic operating conditions, and regeneration requirements. These results refer specifically to the controlled high-loading conditions evaluated in the present study.

In this context, upstream physical pretreatment may represent a useful strategy for reducing the non-target load reaching the adsorption bed. Microfiltration followed by ultrafiltration could remove suspended and colloidal material and part of the higher-molecular-weight organic fraction before adsorption, thereby potentially reducing fouling and competition for accessible interaction domains. The importance of controlling wastewater composition prior to adsorption is supported by studies showing that dissolved organic matter can inhibit micropollutant uptake by β-CD-based adsorbents and that adsorption in other sorbent systems is strongly influenced by the concentration and adsorbability of background dissolved organic carbon [18,29]. Such pretreatment could therefore help preserve adsorption performance and potentially delay adsorbent exhaustion during prolonged operation. However, this benefit was not directly evaluated in the present laboratory-scale study and should be assessed experimentally during subsequent pilot-scale validation.

The relatively high cyproconazole concentration used in the wastewater-matrix experiment was deliberately selected as a controlled high-loading challenge condition rather than to reproduce environmentally occurring concentrations. This approach imposed a sufficiently high contaminant mass loading on the polymer during the short-duration prototype experiments and allowed the influence of the aqueous matrix to be evaluated under a demanding continuous-flow condition. However, individual CECs in treated municipal wastewater generally occur in the ng–µg L^−1^ range and are encountered as complex mixtures during prolonged treatment. The removal efficiencies obtained at mg L^−1^ concentrations should therefore not be extrapolated quantitatively to trace-level treatment conditions.

The competitive adsorption trends observed in the binary and ternary experiments may also depend on the total contaminant loading applied. At environmentally relevant concentrations, occupancy of the accessible adsorption domains, competition among target contaminants, competition with dissolved wastewater constituents, and mass-transfer behavior may differ from those occurring under the present high-loading conditions. Consequently, the relative removal ranking and competitive effects reported here should be interpreted as responses to the tested experimental conditions rather than as validated predictors of performance in municipal wastewater at trace contaminant concentrations. Prolonged fixed-condition experiments using representative wastewater matrices and environmentally relevant multicomponent concentrations will be required to establish whether these trends persist during realistic treatment operation.

### 3.5. Desorption and Post-Desorption Rinsing

The feasibility of recovering the adsorbed contaminant from the β-CD-EPI polymer under continuous-flow conditions was subsequently evaluated using cyproconazole as the model compound. Prior to desorption, the polymer was loaded with a 10 mg L^−1^ cyproconazole solution under continuous-flow conditions, resulting in approximately 92% removal. Desorption with 220 mM acetate buffer at pH 4.0, applied at a desorbent-to-adsorbent ratio of 10 mL g^−1^, recovered approximately 76% of the retained cyproconazole at a nominal contact time of 5 min. Increasing the desorption contact time to 10 min increased recovery to approximately 81%, whereas a further increase to 15 min produced only a marginal improvement, reaching approximately 82%. Thus, most of the recoverable cyproconazole was released within the first 10 min, after which only a marginal additional increase was observed. On a descriptive basis, the 10 min desorption condition was therefore identified as a preliminary practical compromise between contaminant recovery and process duration. Because the tested conditions were evaluated as operational optimization points rather than as independently replicated continuous-flow desorption cycles, the reported recovery values should not be interpreted as statistically validated estimates of desorption reproducibility.

The continuous-flow desorption results provide an experimental link with the regeneration strategy previously established during batch-scale prototype development. In the preceding batch study, desorption of the β-CD-EPI polymer was performed using the same 220 mM acetate buffer at pH 4.0, but with a 30 min contact period and a buffer-to-polymer ratio of 50 mL g^−1^ [20]. Importantly, the reusability of the β-CD-EPI polymer was also demonstrated under batch conditions for at least 10 consecutive adsorption–desorption cycles, maintaining more than 90% of its adsorption capability [20]. Under the present continuous-flow conditions, approximately 81% of the retained cyproconazole was recovered after only 10 min using a desorbent-to-adsorbent ratio of 10 mL g^−1^. Although the target contaminant and operating configuration differed between the batch and continuous-flow experiments, the present results provide preliminary operational evidence that the desorption procedure can be implemented under continuous-flow conditions. Within the tested configuration, cyproconazole recovery above 80% was obtained using a shorter contact time and a lower desorbent-to-adsorbent ratio than those previously applied during batch regeneration. However, this comparison does not establish equivalent regeneration efficiency or reproducibility between batch and continuous-flow operation. Desorption duration and regenerant consumption nevertheless remain relevant variables for subsequent cyclic validation because they directly influence cycle time, secondary liquid-stream generation, and overall operating requirements.

The previously demonstrated reusability of the β-CD-EPI polymer under batch conditions supports its potential for regeneration but does not establish its reusability under continuous-flow operation. The key unresolved question is whether adsorption capacity, desorption efficiency, physical integrity, and hydraulic stability can be maintained over repeated adsorption–desorption cycles in the continuous-flow configuration. The present study addresses an intermediate operational step by identifying preliminary conditions for cyproconazole desorption and subsequent bed rinsing. Repeated adsorption–desorption cycles were not evaluated under continuous-flow conditions; therefore, cyclic stability and long-term regeneration performance must be established in future fixed-condition experiments.

Regenerability has also been demonstrated for other CD-based adsorption systems using different desorption strategies. Romita et al. showed that a water-insoluble β-CD-EPI polymer used for sulfamethoxazole adsorption could be regenerated with 0.5 M sodium bromide without loss of adsorption performance [30]. Chemically distinct styrenic β-CD polymers have also been subjected to solvent-based regeneration during laboratory-scale wastewater experiments [28]. More recently, long-term pilot operation of StyDex columns treating municipal wastewater demonstrated effective regeneration following bed exhaustion, with a substantial fraction of retained contaminants recovered during the initial desorption stage [31]. Although the adsorbates, polymer chemistries, regenerants, and operating configurations differ among these studies, collectively, they demonstrate the technical relevance of incorporating regeneration into the development of cyclodextrin-based wastewater-treatment processes. The reported regeneration of β-CD-EPI with 0.5 M sodium bromide without loss of adsorption performance further supports the intrinsic reusability of this class of polymeric adsorbents [30].

Following desorption, an effective rinsing step was required to displace the residual acetate buffer and restore the aqueous conditions of the polymer bed before subsequent adsorption use. The comparison of the low-flow rinsing profiles showed that, after 30 min, the effluent pH increased from approximately 4.3 to 6.3 at 2 BV h^−1^, whereas final values of approximately 7.1–7.2 were reached at 3–5 BV h^−1^. The lowest flow rate was retained for the initial stage to promote gradual buffer displacement while limiting the hydraulic load applied to the hydrated polymer bed. A subsequent 10 min high-flow rinse increased the effluent pH from approximately 6.3 to 6.8 at 10 BV h^−1^, while increasing the flow rate to 20–40 BV h^−1^ produced only a limited additional improvement, with final pH values of approximately 6.9–7.0. Therefore, 10 BV h^−1^ was selected as the lowest high-flow condition capable of restoring the effluent to the near-neutral operational range. The resulting two-stage procedure comprised 30 min at 2 BV h^−1^ (6 L h^−1^), followed by 10 min at 10 BV h^−1^ (30 L h^−1^), with a total rinse-water requirement of approximately 2.67 BV. This sequence was selected as a preliminary operational compromise between buffer displacement, hydraulic loading, rinsing duration, and recovery of the effluent pH, rather than as a statistically optimized rinsing protocol.

**Figure 5:**
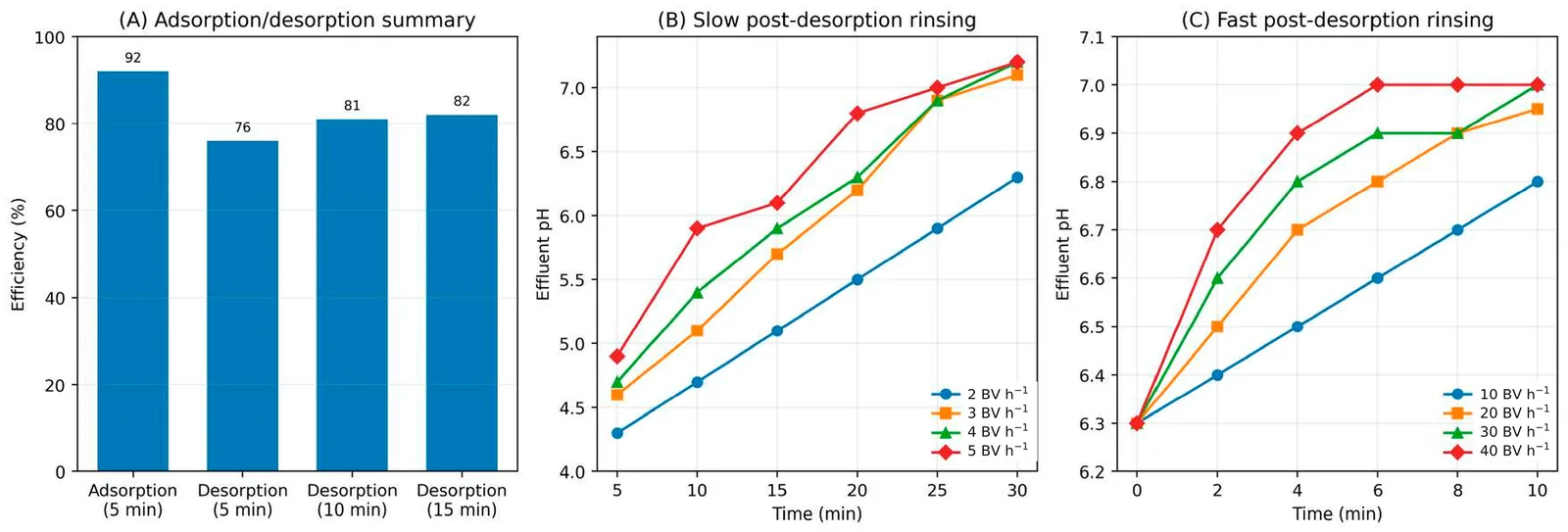
Desorption and post-desorption rinsing behavior of the β-CD-EPI polymer under continuous-flow conditions. (**A**) Summary of cyproconazole adsorption and desorption efficiencies, showing the reference adsorption-loading step and desorption efficiencies obtained with 220 mM acetate buffer at pH 4.0 at nominal contact times of 5, 10, and 15 min. (**B**) Evolution of effluent pH during slow post-desorption rinsing with demineralized water at 2, 3, 4, and 5 BV h^−1^. (**C**) Evolution of effluent pH during fast post-desorption rinsing with demineralized water at 10, 20, 30, and 40 BV h^−1^. Based on these quantitative pH profiles, the selected preliminary rinsing sequence comprised 30 min at 2 BV h^−1^ followed by 10 min at 10 BV h^−1^, corresponding to a total rinse-water requirement of approximately 2.67 BV, which restores the effluent to the near-neutral operational range. The desorption efficiencies and effluent-pH profiles are presented as operational screening data without dispersion statistics and do not represent mean values obtained from independently replicated continuous-flow cycles.

Maintaining the hydrated state of the β-CD-EPI bed during the transition from desorption to rinsing was also operationally important. Prolonged drainage of the column could promote compaction of the hydrated polymer and thereby compromise subsequent hydraulic behavior. Post-desorption rinsing should therefore be considered an integral component of the operating sequence rather than a secondary washing step. The desorption response and the evolution of effluent pH during the subsequent slow- and fast-rinsing stages are shown in Figure 5.

Overall, the present experiments establish preliminary operational conditions for a continuous-flow desorption and rinsing sequence rather than demonstrating cyclic regeneration under continuous-flow operation. Acetate-buffer treatment recovered more than 80% of the retained cyproconazole within 10 min in the tested operational sequence, while the subsequent two-stage water rinse restored the effluent pH to the operating range. The experiments also highlighted the need to preserve the hydrated state of the polymer bed throughout the desorption–rinsing transition to minimize compaction and maintain hydraulic performance. When considered together with the previously demonstrated batch-scale reusability of the polymer [20,30], these results provide a basis for subsequent replicated cyclic continuous-flow experiments. Such studies will be required to determine the variability of desorption recovery and confirm whether adsorption capacity, desorption efficiency, polymer integrity, and hydraulic stability can be maintained over repeated cycles before transferring these conditions to larger reactor configurations.

### 3.6. Operational Implications and Requirements for Subsequent Process Development

The present continuous-flow validation demonstrates that batch-derived adsorption and material parameters provide a necessary starting point for process development but are not sufficient, by themselves, to define the final operating conditions of a dynamic β-CD-EPI adsorption system. The previous batch-based study established the prototype architecture, adsorbent-volume range, column dimensions, preliminary operating conditions, and polymer regeneration strategy required for construction of the laboratory-scale unit [20]. However, experimental operation of the hydrated β-CD-EPI bed revealed additional constraints that could not be identified from batch experiments alone, particularly the strong coupling between polymer swelling, bed compression, flow direction, and hydraulic resistance. Stable operation could not be achieved simply by increasing column diameter or modifying polymer loading under downflow conditions. Instead, the operating configuration had to be adapted to the physical behavior of the hydrated polymer by using the 90 mm column in upflow mobile-bed mode and maintaining the superficial linear velocity below a conservative, configuration-specific practical upper boundary of approximately 12 m h^−1^. Thus, the first requirement for translating the batch-derived design into a functional continuous process was the experimental identification of a hydrodynamic operating range compatible with the swelling and compressibility of the β-CD-EPI bed.

Once hydraulic stability had been established, the adsorption experiments revealed a second limitation of direct batch-to-flow extrapolation: equilibrium adsorption capacity and batch kinetic parameters did not translate proportionally into dynamic removal efficiency. To make the batch-to-column mapping explicit, at the same initial concentration subsequently used in the continuous-flow experiments (*C*_0_ = 5 mg L^−1^), the batch experimental equilibrium capacities (q_e,exp_) were measured and found to be 0.126 mg g^−1^ for furosemide and 0.106 mg g^−1^ for hydrochlorothiazide. The corresponding pseudo-second-order rate constants (k_2_) were 3.691 and 7.322 g mg^−1^ min^−1^, with R^2^ values of 0.998 and 0.992, respectively [20]. Across the complete batch concentration range, furosemide exhibited a higher Langmuir-derived maximum adsorption capacity than hydrochlorothiazide (1.282 versus 0.844 mg g^−1^) and reached apparent equilibrium within approximately 10–20 min, compared with 40–60 min for hydrochlorothiazide [20]. These batch-derived parameters, considered together with polymer density, swelling behavior, and the process-design constraints established in the preceding study, informed the selection of the preliminary polymer inventory and nominal contact-time range for experimental column evaluation. They were used as initial material-performance and design inputs rather than as direct predictors of column removal. Indeed, under continuous-flow conditions, furosemide removal remained below 30% across the three coupled hydraulic conditions, whereas hydrochlorothiazide removal reached approximately 40–65%. These results should not be interpreted as an independent effect of nominal contact time because superficial linear velocity varied simultaneously. More broadly, the five-contaminant evaluation showed that dynamic removal varied markedly among compounds, ranging from consistently >90% for cyproconazole to substantially lower values for furosemide and ciprofloxacin. These findings demonstrate that batch-derived parameters are useful for establishing material feasibility and preliminary process sizing, but contaminant-specific dynamic performance must be validated experimentally because the effective removal achieved under flow reflects the combined influence of apparent affinity, mass-transfer conditions, and reactor hydrodynamics. This staged interpretation is consistent with adsorption-process frameworks in which batch equilibrium and kinetic characterization and continuous-flow behavior represent complementary rather than interchangeable levels of process assessment [14].

A third requirement for subsequent process development is the incorporation of multicomponent competition and wastewater-matrix effects into performance assessment. High removal in single-solute experiments did not guarantee equivalent behavior as aqueous-system complexity increased. Cyproconazole, for example, showed >90% removal when evaluated individually in tap water, whereas its removal decreased to approximately 48–74% in the ternary contaminant mixture prepared in the same aqueous matrix and to approximately 48–55% when evaluated at the same influent concentration in secondary-treated wastewater. Multicomponent exposure produced even larger relative effects on acetaminophen and ciprofloxacin. These observations demonstrate that adsorption performance depends not only on contaminant identity and reactor hydrodynamics, but also on influent composition, combined solute loading, and the physicochemical characteristics of the aqueous matrix. Consequently, subsequent process development should include prolonged fixed-condition validation using representative wastewater matrices, environmentally relevant multicomponent concentrations, and conventional breakthrough assessment before polymer loading and operating conditions are established for larger reactor configurations.

Mechanistic characterization represents an additional requirement for subsequent development of the adsorption system. The compound-dependent removal efficiencies, the preserved retention order in multicomponent experiments, the decrease in removal as mixture complexity increased, and the pronounced effect of the wastewater matrix provide process-level evidence consistent with contaminant-specific affinity and competition for partially overlapping adsorption environments within the β-CD-EPI network. The batch equilibrium and intraparticle-diffusion results obtained for the polymer evaluated in the present study [20], and the molecular-modeling results obtained for a related β-CD-EPI polymer system [10] support the combined contribution of host–guest association with the β-CD cavities, interactions with the heterogeneous EPI-cross-linked network, and diffusion through the hydrated polymer. However, the present continuous-flow experiments were not designed to isolate these contributions or demonstrate a unique molecular mechanism. Direct quantification of the relative importance of cavity inclusion, hydrogen bonding, hydrophobic association, electrostatic effects, and polymer-network diffusion will require targeted spectroscopic, calorimetric, competitive-displacement, and molecular-simulation studies under controlled solution conditions.

A fourth operational requirement concerns regeneration, which should be incorporated into process development together with adsorption rather than considered as an independent downstream operation. Reusability of the β-CD-EPI polymer had previously been demonstrated over repeated batch adsorption–desorption cycles [20], whereas the present experiments identified preliminary conditions for implementing desorption and rinsing in the continuous-flow configuration. In the tested operational sequence, more than 80% of the retained cyproconazole was recovered using 220 mM acetate buffer at pH 4.0 within 10 min, after which a two-stage water rinse restored the column effluent to the operating pH range. These recoveries and rinsing values were obtained as operational optimization data without dispersion statistics and should not be interpreted as evidence of reproducible cyclic performance. The experiments also identified the need to maintain the β-CD-EPI bed in a hydrated state during the transition between adsorption, desorption, and rinsing to minimize compaction and preserve flow behavior. Subsequent process development should therefore include replicated continuous-flow adsorption–desorption cycles to quantify recovery variability, capacity retention, polymer integrity, hydraulic stability, regenerant consumption, and rinsing-water demand.

The mapping between the batch-derived design inputs and the corresponding laboratory-scale continuous-flow observations, together with the requirements identified for subsequent process development, is summarized in Table 3.

Taken together, the results support a staged development framework in which batch-derived equilibrium, kinetic, physicochemical, and reusability data are first used to establish an engineering design, which is then refined by experimental validation under continuous-flow conditions. For the β-CD-EPI system, this second stage required selection of an upflow mobile-bed configuration with visually observed bed expansion to control hydraulic resistance, contaminant-specific assessment of dynamic adsorption, evaluation of multicomponent competition and wastewater-matrix effects, and operational integration of desorption and rinsing. Continuous-flow validation therefore does not replace the preceding batch studies but complements them by identifying process constraints that cannot be resolved under equilibrium conditions. Batch-derived parameters should consequently be considered design inputs rather than direct predictors of dynamic treatment performance. The laboratory-scale observations reported here identify the operating conditions and unresolved performance parameters that should be examined before and during subsequent semi-industrial validation under representative wastewater-treatment conditions.

The present study was deliberately designed to identify a hydraulically stable operating range and evaluate the short-term dynamic adsorption response of the β-CD-EPI system before undertaking prolonged fixed-condition operation. The reported C/C_0_ values therefore represent normalized outlet concentrations obtained under distinct coupled nominal contact-time/superficial-velocity conditions and should not be interpreted as time-on-stream breakthrough curves. The system was not operated for an extended period under a single fixed combination of influent concentration, flow rate, polymer loading, and bed geometry, with sequential outlet sampling until bed exhaustion. Consequently, no breakthrough curve, breakthrough time, dynamic adsorption capacity, number of bed volumes treated to breakthrough, or bed service life can be derived retrospectively from these experiments. Extended operation under fixed hydraulic and influent conditions, with sequential sampling until bed exhaustion, will be required to determine conventional breakthrough profiles, mass-transfer behavior, dynamic adsorption capacity, and bed service life [32].

Recent pilot-scale work with a chemically distinct styrene-functionalized β-CD bead adsorbent has similarly demonstrated that optimal operating conditions can be contaminant-dependent and that long-term assessment requires integrated evaluation of breakthrough behavior, competitive sorption, wastewater-matrix effects, and adsorbent regeneration [31]. These findings reinforce the need for cyclic and fixed-condition validation as subsequent steps before the β-CD-EPI process can be translated to larger reactor configurations.

The hydrodynamic operating boundary identified in this study is specific to the tested column geometry, polymer loading, hydrated-bed characteristics, retaining elements, and hydraulic circuit. Although the replicated downflow experiments and direct flow-direction comparison, together with the broader descriptive upflow screening, provided a quantitative basis for comparing the tested configurations, the lack of systematic expanded-bed-height measurements prevented determination of bed expansion ratio, voidage, and minimum fluidization or expansion velocity. These parameters should be determined during subsequent scale-dependent hydrodynamic characterization before transferring the operating boundary to larger reactor geometries.

## 4. Conclusions

This study experimentally validated the transition of a batch-derived β-CD-EPI adsorption process to laboratory-scale continuous-flow operation and demonstrated that successful translation requires consideration of the hydrodynamic behavior of the hydrated polymer bed in addition to its adsorption properties. The batch-derived parameters provided a suitable basis for prototype design, whereas continuous operation revealed additional constraints associated with polymer swelling, bed compressibility, flow direction, and hydraulic resistance. Upflow mobile-bed operation in the 90 mm column provided the greatest hydraulic stability among the tested configurations. Superficial linear velocities below approximately 12 m h^−1^ defined a conservative, configuration-specific operating range based on the combined evidence from replicated downflow experiments, the replicated direct flow-direction comparison, the broader descriptive upflow screening, and qualitative observations of bed mobilization. This value should not be interpreted as a minimum fluidization velocity, and quantitative bed-expansion parameters will need to be determined before transferring this operating range to larger reactor geometries.

Continuous-flow removal was strongly contaminant-dependent and could not be predicted directly from batch-derived equilibrium capacities or equilibration times. This was particularly evident for furosemide and hydrochlorothiazide, whose relative performance under dynamic conditions differed from that anticipated from the preceding batch experiments. Increasing system complexity further reduced adsorption performance: multicomponent exposure decreased the removal of cyproconazole, acetaminophen, and ciprofloxacin, while cyproconazole removal decreased from >90% in tap water to approximately 48–55% in secondary-treated wastewater. The observed contaminant selectivity and the progressive decrease in removal under multicomponent and wastewater-matrix conditions are consistent with heterogeneous and partially overlapping adsorption environments involving the β-CD cavities and the EPI-cross-linked polymer network. However, these process-level observations do not demonstrate a unique molecular interaction mechanism, which will require targeted mechanistic investigation. These results demonstrate that batch-derived parameters should be regarded as design inputs rather than direct predictors of continuous-flow performance and that contaminant competition and wastewater-matrix effects must be incorporated into subsequent process development.

Because the adsorption experiments were conducted at mg L^−1^ concentrations as controlled high-loading challenges, the resulting removal efficiencies and competitive trends should not be extrapolated quantitatively to environmentally relevant concentrations. Their applicability to realistic municipal wastewater treatment must be established through prolonged fixed-condition experiments using representative multicomponent matrices at ng–µg L^−1^ levels.

The study also identified preliminary operating conditions for implementing desorption and rinsing under continuous-flow conditions. In the tested operational sequence, more than 80% of the retained cyproconazole was recovered using 220 mM acetate buffer at pH 4.0 within 10 min, followed by a two-stage water rinse that restored the effluent pH while maintaining the polymer bed in its hydrated state. Because these values were obtained from operational optimization tests without dispersion statistics, they do not demonstrate the reproducibility of desorption or cyclic regeneration under continuous-flow conditions. Together with the previously demonstrated batch-scale reusability of the β-CD-EPI polymer, the present findings provide a basis for subsequent replicated cyclic continuous-flow validation. Extended operation under fixed influent and hydraulic conditions, repeated adsorption–desorption cycles, and prolonged multicomponent exposure will be required to determine recovery variability, retention-capacity loss, conventional breakthrough behavior, dynamic adsorption capacity, bed service life, polymer integrity, hydraulic stability, and long-term process performance before translation to larger reactor configurations.

## Figures and Tables

**Table 1:** Operating conditions used for the continuous-flow adsorption experiments with the β-CD-EPI polymer.

Nominal Contact Time t_C_ (min)	Flow Rate Q (L h^−1^)	Superficial Linear Velocity (m h^−1^)	Hydraulic Loading (BV h^−1^)
3	60	10.7	20
5	36	6.4	12
10	18	3.2	6

Experimental configuration: 90 mm diameter column; β-CD-EPI loading of 705 g; approximate hydrated adsorbent-bed volume of 3 L; bed depth of approximately 0.54 m; upflow mobile-bed operation with visually observed bed expansion.

**Table 2:** Physicochemical characteristics of the secondary-treated effluent used in the wastewater-matrix experiment.

Parameter	Value
BOD_5_	9.36 ± 1.50 mg L^−1^
COD	46.43 ± 4.22 mg L^−1^
Suspended solids	7.37 ± 1.17 mg L^−1^
pH	7.38 ± 0.09
Electrical conductivity	2093 ± 37.64 µS cm^−1^
NH_4_-N	14.35 ± 2.31 mg L^−1^
NO_3_-N	8.79 ± 1.84 mg L^−1^
Total nitrogen	26.5 ± 3.16 mg L^−1^
Total phosphorus	1.56 ± 0.41 mg L^−1^

Values are expressed as mean ± SD. BOD_5_, five-day biochemical oxygen demand; COD, chemical oxygen demand; NH_4_-N, ammonium nitrogen; NO_3_-N, nitrate nitrogen.

**Table 3:** Mapping of batch-derived design inputs to laboratory-scale continuous-flow observations and requirements for subsequent development of the β-CD-EPI polymer adsorption process.

Process Parameter/Requirement	Batch-Derived Input or Continuous-Flow Criterion	Requirement for Subsequent Process Development
Adsorption column configuration	90 mm nominal-diameter column	Provides the laboratory geometry validated under continuous-flow operation
Polymer loading	705 g β-CD-EPI, corresponding to approximately 3 L of hydrated bed and a 0.54 m bed depth	Defines the experimentally validated laboratory-scale polymer inventory
Batch adsorption-capacity input	At *C*_0_ = 5 mg L^−1^, q_e,exp_ was 0.126 mg g^−1^ for furosemide and 0.106 mg g^−1^ for hydrochlorothiazide. Langmuir q_max_ values were 1.282 and 0.844 mg g^−1^, respectively [20]	Used together with polymer density and swelling behavior to inform the preliminary polymer inventory; batch capacity and compound ranking must be confirmed under dynamic operation
Batch kinetic input	At *C*_0_ = 5 mg L^−1^, pseudo-second-order *k*_2_ values were 3.691 and 7.322 g mg^−1^ min^−1^ for furosemide and hydrochlorothiazide, with R^2^ values of 0.998 and 0.992, respectively. Apparent equilibrium was reached within approximately 10–20 and 40–60 min [20]	Informed the initial nominal contact-time range evaluated in the column; batch *k*_2_ and equilibration time cannot be equated directly with column residence time or dynamic mass-transfer behavior
Flow direction	Upflow mobile-bed operation with visually observed bed expansion	Minimizes bed compaction and excessive pressure development
Superficial linear velocity	Practical upper operating limit of approximately 12 m h^−1^	The operating range must be re-evaluated for each larger reactor geometry rather than extrapolated directly from the laboratory configuration
Coupled adsorption operating conditions	3 min/10.7 m h^−1^, 5 min/6.4 m h^−1^ and 10 min/3.2 m h^−1^	Nominal contact time and superficial linear velocity were varied simultaneously and their individual effects cannot be resolved from the present experiments
Dynamic adsorption performance	Strongly contaminant dependent	Batch-derived equilibrium and kinetic parameters should be treated as design inputs rather than direct predictors of continuous-flow removal
Multicomponent operation	Removal decreased relative to single-solute conditions	Polymer requirements should be established using representative contaminant mixtures
Wastewater matrix	Cyproconazole removal decreased from >90% in tap water to approximately 48–55% in secondary-treated wastewater under the tested high-loading conditions	Adsorption performance must be validated using representative wastewater matrices rather than inferred from clean-water experiments
Desorption	220 mM acetate buffer, pH 4.0; 10 mL g^−1^; 10 min; >80% cyproconazole recovery	Regenerant consumption and desorption duration should be incorporated into process sizing
Post-desorption rinsing	30 min at 2 BV h^−1^ (6 L h^−1^; 1.0 BV), followed by 10 min at 10 BV h^−1^ (30 L h^−1^; 1.67 BV); total rinse-water requirement of approximately 2.67 BV; final effluent pH of approximately 6.8	Preliminary sequence selected from the quantitative effluent-pH profiles as a compromise between buffer displacement, hydraulic loading, rinsing duration, and recovery of the near-neutral operating range; rinsing-water demand and the endpoint criterion should be revalidated during scale-up
Bed management	Maintain the β-CD-EPI polymer in the hydrated state during adsorption–desorption–rinsing transitions	Minimizes compaction and helps preserve hydraulic performance
Polymer reusability	Repeated adsorption–desorption reusability previously demonstrated under batch conditions [20]	Provides the material basis for cyclic operation; stability must now be confirmed under repeated continuous-flow cycles
Continuous cyclic validation	Repeated continuous adsorption–desorption cycles not evaluated in the present study	Adsorption capacity, desorption efficiency, polymer integrity, and hydraulic stability require validation under prolonged continuous cyclic operation
Influent loading regime	Present laboratory experiments performed at mg L^−1^ concentrations as controlled high-loading challenge conditions	Removal efficiencies and competitive trends require validation through prolonged fixed-condition experiments using representative multicomponent wastewater at environmentally relevant ng–µg L^−1^ concentrations
Breakthrough characterization	Conventional time-on-stream breakthrough under fixed hydraulic conditions was not determined	Extended operation at constant influent concentration, flow rate, polymer loading, and bed geometry is required to determine breakthrough profiles, dynamic adsorption capacity, bed volumes treated to breakthrough, and bed service life
Subsequent process stage	Laboratory-scale operating range and short-term adsorption response experimentally evaluated	Fixed-condition breakthrough, cyclic stability, and scale-dependent hydrodynamic characterization are required before translation to larger reactor configurations

Note: Desorption and post-desorption rinsing criteria were established using the dedicated 63 mm regeneration configuration containing 100 g of β-CD-EPI polymer and should therefore be revalidated during subsequent process scale-up.

## Data Availability

The original contributions presented in this study are included in the article/Supplementary Materials. Further inquiries can be directed to the corresponding author.

## Supplementary Materials

The following supporting information can be downloaded at https://www.mdpi.com/article/10.3390/polym18182292/s1, Figure S1: Hydrodynamic screening of the β-CD-EPI polymer bed under downflow continuous-flow operation. Pressure-drop evolution as a function of superficial linear velocity in the 63 mm column containing (A) 235 g, (B) 470 g and (C) 705 g of β-CD-EPI polymer, and in the 90 mm column containing (D) 235 g, (E) 470 g and (F) 705 g of β-CD-EPI polymer. Data are presented as mean ± SD (n = 3); Figure S2: Hydrodynamic behavior of the β-CD-EPI polymer bed under upflow mobile-bed operation with visually observed bed expansion. Pressure-drop evolution as a function of superficial linear velocity in the 90 mm column containing (A) 470 g and (B) 705 g of β-CD-EPI polymer. Superficial linear velocities of 4, 8, 12, 16 and 20 m h^−1^ were evaluated. The pressure-drop profiles provide quantitative operational data for identifying a configuration-specific practical operating range, whereas bed expansion and particle mobility were assessed qualitatively by visual observation. These profiles are presented as descriptive operational screening data and do not represent mean values derived from independently replicated experiments; Table S1: MRMHR acquisition parameters used for the determination of the five target contaminants by UHPLC-HRMS/MS.

## Abbreviations

The following abbreviations and symbols are used in this manuscript:

Abbreviation	Definition
BOD_5_	Five-day biochemical oxygen demand
BV	Bed volume
CD	Cyclodextrin
CEC	Contaminant of emerging concern
COD	Chemical oxygen demand
EPI	Epichlorohydrin
MRMHR	Multiple-reaction-monitoring high-resolution acquisition mode
NH_4_-N	Ammonium nitrogen
NO_3_-N	Nitrate nitrogen
PES	Polyethersulfone
PVC	Polyvinyl chloride
QTOF	Quadrupole time-of-flight mass spectrometry
SD	Standard deviation
UHPLC-HRMS/MS	Ultra-high-performance liquid chromatography coupled to high-resolution tandem mass spectrometry
WWTP	Wastewater treatment plant
β-CD	β-Cyclodextrin
β-CD–EPI	Water-insoluble β-cyclodextrin-epichlorohydrin polymer
Symbol	Definition
C_0_	Influent contaminant concentration
C	Effluent contaminant concentration
C/C_0_	Normalized effluent concentration
Q	Volumetric flow rate
t_c_	Nominal contact time
V_ads_	Hydrated adsorbent-bed volume
Δp	Pressure drop across the adsorbent bed
