# Supplementary material for: Batch-to-Flow Translation of β-Cyclodextrin Polymer Adsorption for Emerging Contaminant Removal: Hydrodynamic and Operational Validation
Source: Polymers (Basel). 2026 Sep 19;18(18):2292. doi: 10.3390/polym18182292 (PMC13611648; doi:10.3390/polym18182292)
Supplement: Supplementary file 1 [file polymers-18-02292-s001.zip › polymers-4546912-supplementary.pdf]

# Batch-to-Flow Translation of β-Cyclodextrin Polymer Adsorption for Emerging Contaminant Removal: Hydrodynamic and Operational Validation

**Antonio Tomás Hernández Cegarra[1], Teresa Gómez-Morte[1], José Antonio Pellicer[1], María Isabel Rodríguez-López[1], Nuria Vela[2], Ángel Gil-Izquierdo[3], Estrella Núñez-Delicado[1] and José Antonio Gabaldón[1],***

[1] Molecular Recognition and Encapsulation Research Group (REM), Health Sciences Department, Universidad Católica de Murcia (UCAM), Campus de los Jerónimos 135, E-30107 Guadalupe, Murcia, Spain; athernandez@alu.ucam.edu (A.T.H.C.); tgomez@ucam.edu (T.G.-M.); japellicer@ucam.edu (J.A.P.); mirodriguez@ucam.edu (M.I.R.-L.); enunez@ucam.edu (E.N.-D.)

[2] Applied Technology Group to Environmental Health, Universidad Católica de Murcia (UCAM), Campus de los Jerónimos 135, E-30107 Guadalupe, Murcia, Spain; nvela@ucam.edu (N.V.)

[3] Research Group on Quality, Safety, and Bioactivity of Plant-Derived Foods, Department of Food Science and Technology, Centro de Edafología y Biología Aplicada del Segura–Consejo Superior de Investigaciones Científicas (CEBAS-CSIC), Campus de Espinardo 25, E-30100 Murcia, Spain; angelgil@cebas.csic.es (A.G.)

* Correspondence: jagabaldon@ucam.edu (J.A.-G.); Tel.: +34-968-278622

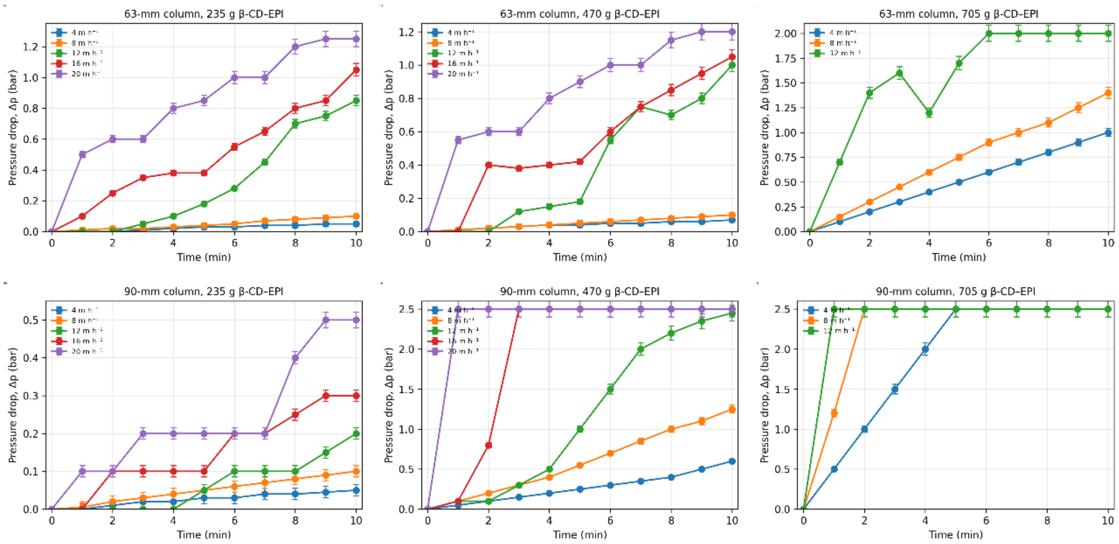


**Figure S1**. Hydrodynamic screening of the β-CD-EPI polymer bed under downflow continuous-flow operation. Pressure-drop evolution as a function of superficial linear velocity in the 63 mm column containing (A) 235 g, (B) 470 g and (C) 705 g of β-CD-EPI polymer, and in the 90 mm column containing (D) 235 g, (E) 470 g and (F) 705 g of β-CD-EPI polymer. Data are presented as mean ± SD (n = 3).

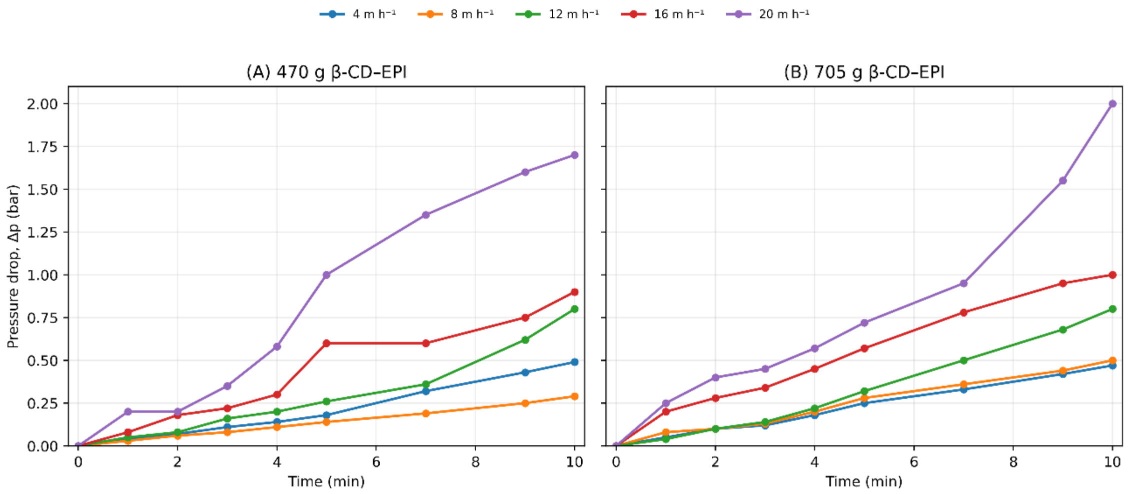


**Figure S2.** Hydrodynamic behavior of the β-CD-EPI polymer bed under upflow mobile-bed operation with visually observed bed expansion. Pressure-drop evolution as a function of superficial linear velocity in the 90 mm column containing (A) 470 g and (B) 705 g of β-CD-EPI polymer. Superficial linear velocities of 4, 8, 12, 16 and 20 m $h^{-1}$ were evaluated. The pressure-drop profiles provide quantitative operational data for identifying a configuration-specific practical operating range, whereas bed expansion and particle mobility were assessed qualitatively by visual observation. These profiles are presented as descriptive operational screening data and do not represent mean values derived from independently replicated experiments.

**Table S1.** MRMHR acquisition parameters used for the determination of the five target contaminants by UHPLC-HRMS/MS.

| Analyte | Ionization mode | Precursor ion (m/z) | Quantifier product ion (m/z) | Qualifier product ion (m/z) |
|---|---|---|---|---|
| Furosemide | ESI− | 329.0004 | 285.0115 | 204.9847 |
| Hydrochlorothiazide | ESI− | 295.9572 | 268.9476 | 204.9854 |
| Acetaminophen | ESI+ | 152.0706 | 110.0721 | 65.0404 |
| Ciprofloxacin | ESI+ | 332.1405 | 288.1507 | 245.1085 |
| Cyproconazole | ESI+ | 292.1211 | 70.0400 | 125.0150 |
